# Comprehensive analysis of disulfidptosis-related genes reveals the effect of disulfidptosis in ulcerative colitis

**DOI:** 10.1038/s41598-024-66533-9

**Published:** 2024-07-08

**Authors:** Huixian Song, Fengrui Zhang, Xinyu Bai, Hao Liang, Junkun Niu, Yinglei Miao

**Affiliations:** 1 Department of Gastroenterology, The First Affiliated Hospital of Kunming Medical University, Kunming, 650032 Yunnan China; 2 Yunnan Province Clinical Research Center for Digestive Diseases, Kunming, 650032 Yunnan China

**Keywords:** Ulcerative colitis, Disulfidptosis, Machine learning, Weighted gene co-expression network analysis, Immune infiltration, Ulcerative colitis, Diagnostic markers, Machine learning

## Abstract

Ulcerative colitis (UC) is a chronic inflammatory condition of the intestinal tract. Various programmed cell death pathways in the intestinal mucosa are crucial to the pathogenesis of UC. Disulfidptosis, a recently identified form of programmed cell death, has not been extensively reported in the context of UC. This study evaluated the expression of disulfidptosis-related genes (DRGs) in UC through public databases and assessed disulfide accumulation in the intestinal mucosal tissues of UC patients and dextran sulfate sodium (DSS)-induced colitis mice via targeted metabolomics. We utilized various bioinformatics techniques to identify UC-specific disulfidptosis signature genes, analyze their potential functions, and investigate their association with immune cell infiltration in UC. The mRNA and protein expression levels of these signature genes were confirmed in the intestinal mucosa of DSS-induced colitis mice and UC patients. A total of 24 DRGs showed differential expression in UC. Our findings underscore the role of disulfide stress in UC. Four UC-related disulfidptosis signature genes—SLC7A11, LRPPRC, NDUFS1, and CD2AP—were identified. Their relationships with immune infiltration in UC were analyzed using CIBERSORT, and their expression levels were validated by quantitative real-time PCR and western blotting. This study provides further insights into their potential functions and explores their links to immune infiltration in UC. In summary, disulfidptosis, as a type of programmed cell death, may significantly influence the pathogenesis of UC by modulating the homeostasis of the intestinal mucosal barrier.

## Introduction

As an important type of inflammatory bowel disease (IBD), ulcerative colitis (UC) has poor treatment efficacy due to its pathogenesis and mechanism have not been fully elucidated. An imbalance in intestinal mucosal barrier homeostasis is one of the important mechanisms leading to the occurrence and development of UC^1^. Previous studies have revealed notable changes for the levels of various classical programmed cell death pathways in UC intestinal epithelial cells (IECs) and intestinal mucosal immune cells, such as apoptosis, necrosis, and pyroptosis, which in turn lead to an imbalance of the intestinal mucosal barrier and immune homeostasis in patients with UC, and are an important influence on the development of UC (Supplementary Table S1-S3)^2,3^. Ferroptosis and cuproptosis have been successively discovered as novel modes of programmed cell death in recent years^4,5^, and their roles and mechanisms in UC are being successively reported, further revealing the key regulatory roles of novel modes of programmed cell death in UC intestinal mucosal homeostasis^6–8^. Therefore, further exploration of the changes and regulatory mechanisms of various types of programmed cell death in UC intestinal mucosal tissues will be beneficial for further elucidating the pathogenesis of UC and finding new therapeutic targets.

As the latest type of programmed cell death that was reported in early 2023^9^, the importance of disulfidptosis is still uncertain, and its role and regulatory mechanisms in UC are still unclear. Disulfidptosis is the newest programmed cell death in which intracellular nicotinamide adenine dinucleotide phosphate (NADPH) is insufficient to reduce excessive disulfides such as cystine under glucose starvation conditions, which leads to intracellular disulfide accumulation and disulfide stress, resulting in rapid cell death^9^. Disulfide stress induced by disulfide accumulation is the key factor leading to disulfidptosis. Studies have shown that glucose metabolism in the intestinal epithelial cells of UC is dramatically enhanced and glucose consumption is significantly increased due to the persistence of chronic inflammation^10^. Moreover, due to the restricted dietary structure, intestinal mucosal damage leads to nutrient absorption disorder and long-term diarrhea, and the intake and absorption of glucose in patients with UC are decreased significantly. The above factors suggest that a relative lack of glucose or even glucose starvation may exist in the intestinal mucosal tissues of UC patients, which is the critical basis for disulfidptosis in the UC intestinal mucosa. It has been reported in the literature that the expression of SLC3A2, which is a chaperone protein that together with SLC7A11 constitutes the glutamate-cystine countertransport system (system XC-) and participates in the intracellular transport of cystine, is upregulated in UC^10–14^, and the content of cystine in the intestinal mucosa of UC is significantly elevated compared with that of normal mucosa^15^. This result suggests that there may be an excessive accumulation of cystine in the UC intestinal mucosa, resulting in a relative deficiency of intracellular NADPH, which is unable to completely reduce the excessive cystine, leading to the accumulation of disulfides and the occurrence of disulfide stress, which is also a key condition for the occurrence of disulfidptosis in the UC intestinal mucosa. In addition, SLC7A11 is involved in the regulation of Treg proliferation and functional phenotype^16^. In tumor diseases, IFN-γ released by T cells inhabits the expression level of the xCT transporter, whereas down-regulation of FLNA expression enhances the resistance of CD8 + cytotoxic T cells (CTLs) to perforin^17^. Treg cells and CTLs play an important role in the alleviation and exacerbation of intestinal inflammation in UC, but the relationship between bisulfide death and immune infiltration of UC and the molecular mechanisms are currently not clear^18,19^. Therefore, it is necessary to clarify the relationship between disulfidptosis and UC and to explore its role and potential regulatory mechanisms in the intestinal mucosa of UC.

**Figure 1:**
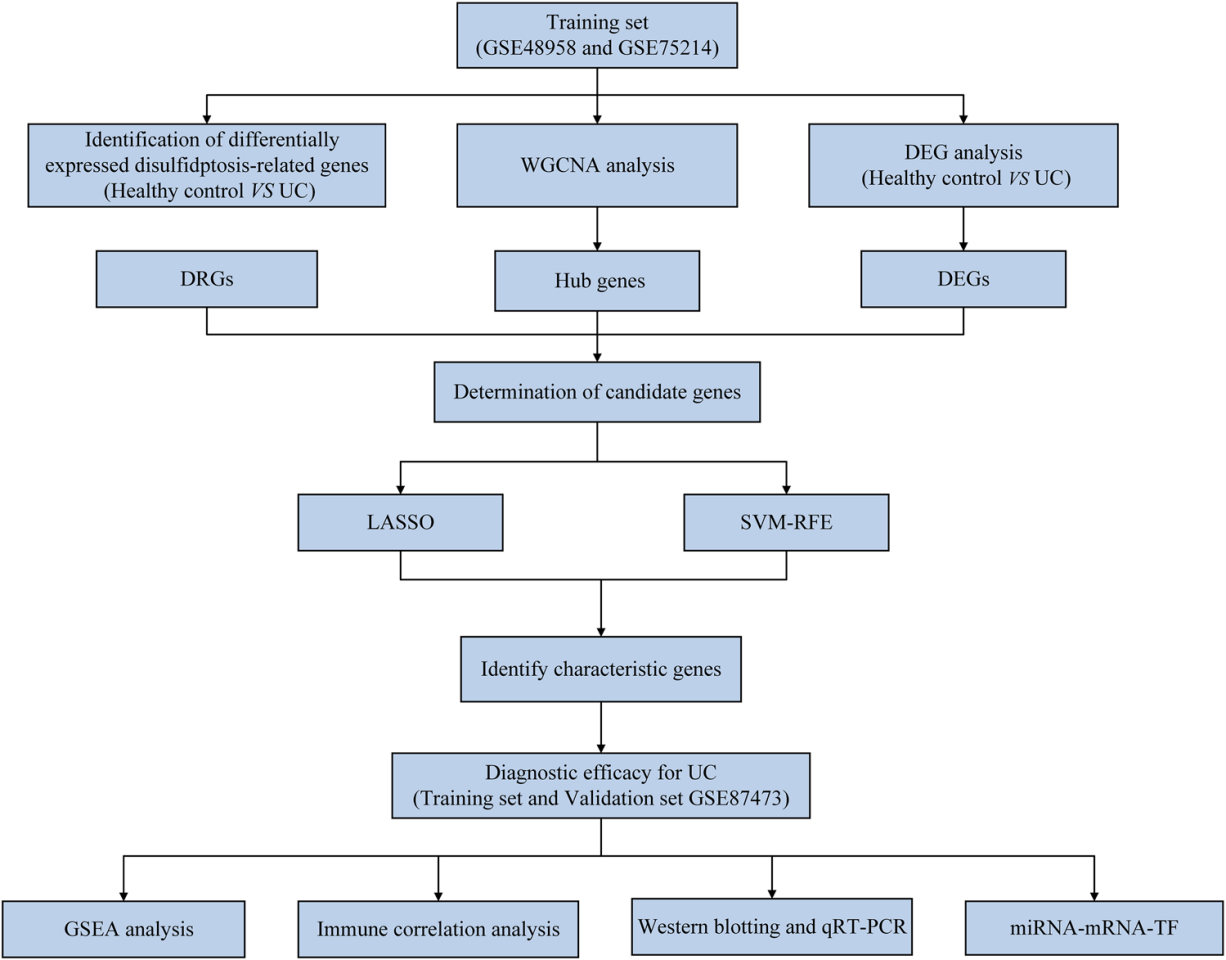
The study flowchart of whole experiment. (Definition of Abbreviations: ulcerative colitis (UC), weighted gene co-expression network analysis (WGCNA), disulfidptosis-related genes (DRGs), differentially expressed genes (DEGs), least absolute shrinkage and selection operator (LASSO), support vector machine-recursive feature elimination (SVM-RFE), gene set enrichment analysis (GSEA), quantitative real time polymerase chain reaction (qRT-PCR), transcription factor (TF).

First, this study assessed the expression of disulfidptosis-related genes (DRGs) in UC using public databases and then detected the disulfide content in the intestinal mucosal tissues of UC patients and dextran sulfate salt (DSS)-induced colitis mice by HPLC–MS-based targeted metabolomics to clarify disulfide accumulation and stress in UC. In summary, the basis for disulfidptosis in the intestinal mucosa of UC was determined. Then, various bioinformatics techniques were utilized to identify UC-related disulfidptosis signature genes, analyze their possible functions in UC, and explore their correlation with immune infiltration in UC. Finally, quantitative real-time PCR (qRT-PCR) and western blotting (WB) were performed to validate the mRNA and protein expression levels of the signature genes in the intestinal mucosal tissues of DSS-induced colitis mice and UC patients. This study showed the potential existence of disulfidptosis in UC and provided a new perspective to elucidate the role and regulatory mechanisms of disulfidptosis in UC. The flow chart of this study is shown in Fig. 1.

## Materials and methods

### Acquisition of datasets and DRGs

The datasets GSE48958, GSE75214, and GSE87473 were downloaded from the GEO public database (https://www.ncbi.nlm.nih.gov/geo/). The GSE48958 dataset contained colon tissue biopsy samples from eight healthy individuals and seven UC patients, and the GSE75214 dataset contained colon tissue biopsy samples from 11 healthy individuals and 74 UC patients. Both datasets were annotated by the GPL6244 [HuGene-1_0-st] Affymetrix Human Gene 1.0 ST Array [transcript (gene) version] platform, merged by batch-effects processing and used as a training set, which contained 19 healthy control samples and 81 UC samples for subsequent analysis. The GSE87473 dataset was used as a validation set, containing 21 healthy control samples and 106 UC samples, to verify the expression and diagnostic value of the signature genes.

Twenty-eight DRGs were obtained according to the published literature (PMID: 36747082), specifically SLC7A11, SLC3A2, NCKAP1, WASF2, CYFIP1, ABI2, BRK1, NUBPL, NDUFA11, LRPPRC, OXSM, NDUFS1, GYS1, FLNA, FLNB, MYL6, MYH9, MYH10, ACTB, ACTN4, CAPZB, CD2AP, DSTN, TLN1, INF2, PDLIM1, IQGAP1 and RPN1 (Supplementary Table S4)^9^.

### Human colonic tissue samples

The study was approved by the Ethics Committee of the First Affiliated Hospital of Kunming Medical University (2023L113). All study participants signed the informed notification of clinical research and consent form before enrollment. We included five UC patients, endoscopically and pathologically confirmed, aged 18 to 60 years, Mayo Endoscopic Score (MES) ≥ 2. These patients had received only mesalazine in the preceding three months and were upgraded to additional treatments post-colonoscopy and sampling, as required. Five healthy controls were also included; these individuals underwent colonoscopy primarily for colorectal tumor screening. Exclusion criteria were adopted from Pei J. et al.^20^. A senior gastroenterologist with expertise in IBD endoscopy performed all colonoscopies for both UC patients and healthy controls. Endoscopic findings were meticulously recorded based on the UC endoscopic scoring system. From each participant, six intestinal mucosal specimens were collected from the sigmoid colon. One sample from each participant was used for histopathological examination, conducted by two pathologists, while the remaining tissues were preserved at − 80 °C.

### Animal experiments

The animal experimental protocol of this study was conducted under the approval of the Laboratory Animal Ethics Committee of Kunming Medical University (KMMU2021186), and the experimental mice were obtained from the Department of Laboratory Animal Science of Kunming Medical University. The study was performed in compliance with the National Guidelines for the Care and Use of Animals. The study was reported in accordance with ARRIVE guidelines. Male C57BL/6J mice aged 6–8 weeks and weighing 18–22 g were selected for the experiments, and all mice were housed in an specific pathogen free (SPF) environment. The mice were randomly divided into control and DSS-treated groups of ten mice each, and the mice in the control group were given distilled water ad libitum for seven days, while the mice in the DSS-treated group were given DSS solution (MP Biomedicals, United States) at a concentration of 2 % ad libitum for seven days. The body weight, fecal characteristics, blood in stool, and DAI scores of the mice were recorded daily^21^. The mice were decapitated on the 8th day of modeling, and the colonic tissues were dissected and stored in a freezer at -80 °C.

### Quantification of cystine, cysteine, glutathionyl-cysteine, glutathione and glutathione disulfide levels by HPLC–MS

An appropriate amount of sample was added to 400 μL of precooled (4 °C) methanol–water (v/v = 4:1, containing 0.1 % formic acid and 1 mM BHT, containing internal standard succinate-2,2,3,3-d4). Two steel beads were added, and the mixture was placed in a freezer at -20 °C for 2 min, placed into a grinder for grinding (60 Hz, 2 min), vortexed for 1 min, sonicated in an ice-water bath for 10 min, and centrifuged for 10 min (4 °C, 12,000 rpm). After 10 min (4 °C, 12,000 rpm), 300 μL of supernatant was evaporated. Then, 300 μL of precooled (4 °C) methanol–water (v/v = 4:1, containing 0.1 % formic acid, 1mM BHT, containing internal standard succinic acid-2,2,3,3-d4) was added to the residue of the supernatant; vortexing was performed for 1 min, sonication was carried out in an ice-water bath for 10 min, and the supernatant was centrifuged for 10 min (4 °C, 12,000 rpm), vortexed for 1 min, sonicated for 10 min, and centrifuged for 10 min (4 °C, 12,000 rpm). Then, 300 μL of supernatant was evaporated, the supernatant was resolubilized with 200 μL of 95 % aqueous acetonitrile (containing L-2-chlorophenylalanine), vortexed for 30 s, sonicated for 5 min in an ice-water bath, and centrifuged for 5 min (4 ℃, 13,000 pm). The supernatant was drawn up by a syringe, filtered by using a hydrophilic syringe with a 0.22-μm filter, transferred to a brown injection vial, and analyzed by LC–MS/MS. Liquid chromatography was performed on an ACQUITY UPLC system (waters Corp). A Waters ACQUITY UPLC HSS PFP column (100 mm × 2.1 mm, 1.8 μm) was used for analysis. The injection volume was 5 μL, and mobile phase A was 0.1 % formic acid–water solution. The mobile phase B was acetonitrile. The gradient conditions were as follows, and the gradient elution was performed at a flow rate of 0.3 mL/min. All samples were kept at 4 °C during analysis and the column temperature was set at 40 °C. Mass spectrometry analysis was performed on an AB Sciex Qtrap 6500 + system. An electrospray ionization (ESI) source was used, operating in positive and negative ion mode. Air curtain gas was used as the collision gas. Additional instrument parameters were as follows: positive ion mode: CUR: 35 Psi, EP: 10 V, 4500 V; ion source temperature: 450 °C; spray gas (Gas 1): 55 Psi; auxiliary heating gas (Gas 2): 60 Psi; negative ion mode: CUR: 35 Psi; EP: -10 V, -4500 V; ion source temperature: 450 °C. Data acquisitions and further analysis were conducted using Analyst software. Data acquisition and subsequent analyses were performed using Analyst software, while SCIEX OS-MQ software was utilized for quantifying all metabolites.

### Construction of the coexpression network and screening of the hub genes

To find modular genes in the training set that are highly correlated with phenotype, weighted gene co-expression network analysis (WGCNA) was performed on all genes using the UC and healthy controls as phenotypes. The samples were first clustered, and outliers were removed. The soft threshold for the data was determined (power = 8, R^2^ = 0.85) to ensure that gene interactions maximally conformed to a scale-free distribution. Then, we constructed a coexpression matrix, calculated the proximity and similarity between genes to construct a systematic clustering tree of genes and identified gene modules by hierarchical clustering. MEDissThres was set to 0.3 to merge similar modules analyzed by the dynamic shear tree algorithm, and the most relevant modules (*p* value < 0.05 and correlation coefficient of 0.4) were screened with UC and healthy controls as key modules. The hub genes were obtained by combining the genes of the key modules.

### Screening of candidate genes and their correlation analysis

Differentially expressed genes (DEGs) between UC samples and healthy control samples were analyzed in the training set using the limma package (adj. *p* value < 0.05 and |log2-fold change (FC) |> 0.5)^22^. The intersection of DEGs, hub genes, and DRGs was taken to obtain candidate genes using the R package UpSetR. The correlation between the intersected genes was calculated in the training set by the Pearson algorithm to obtain the corresponding *p* value and correlation coefficients.

### Screening of feature genes and functional enrichment analysis

The least absolute shrinkage and selection operator (LASSO) algorithm and support vector machine (SVM) algorithm were used to identify the candidate genes in the training set. The R package “Venn Diagram” was used to intersect the genes identified by the two algorithms to obtain the feature genes, and then the R package “*p* ROC” was used to analyze the ROC curves of the genes and to verify their diagnostic value. The R package “ggplot2” was further used to analyze the expression of the signature genes in the UC samples and healthy control samples in the training and validation sets by the Wilcox test method. In addition, gene set enrichment analysis (GSEA) software (V4.0.3) was used for gene set enrichment analysis to explore the possible biological functions of the characterized genes.

### Evaluation of immune infiltration

To investigate immune cell infiltration in UC samples and healthy control samples, the cell type identification by estimating relative subsets of RNA transcripts (CIBERSORT) algorithm and LM22 gene set were used in this study to calculate the proportion of 22 immune cells in all samples in the training set. Differentially abundant immune cells were screened out by the Wilcox test (*p* < 0.05) and visualized by using the R package “ggplot2” to plot the box line plots^23,24^. Then, the correlations among the characteristic genes and the differentially abundant immune cells were analyzed by Spearman’s method.

### Western blotting analysis

Western blotting was performed as described previously^25^. The primary antibodies and concentrations used in this study were anti-vinculin (1:5000, Sigma-Aldrich), anti-SLC7A11 (1:1000, Abmart), anti-LRPPRC (1:5000, Proteintech), anti-NDUFS1 (1:5000, Proteintech), and anti-CD2AP (1:5000, Sigma-Aldrich).

### Quantitative real time polymerase chain reaction

The mRNA expression levels of the characterized genes in the intestinal mucosa of DSS-induced mice and UC patients were examined by qRT-PCR experiments. First, total RNA of colon tissues was isolated and extracted using TRIzol reagent, and cDNA was obtained by reverse transcription using the PrimeScript RT Reagent Kit (Takara, China) and then extracted by an ABI 7500 fluorescence quantitative PCR instrument and Takarat ®SYBR Premix ExTaq Kit for PCR experiments. β-Tubulin was used as an internal reference for PCR amplification and detection. Detection values were normalized using the 2^−△△CT^ method. The specific primer sequences are shown in Table 1**.**

**Table 1:** The primer sequence used in this study.

Genes	Organisms	Forward primer	Reverse primer
SLC7A11	Mus musculus	ACAGGATTGGAAACACACTGCCTA	CAACATCGCAATGCCATGGTA
LRPPRC	Mus musculus	GAGAATGGTGGCTGGACTTGAC	TTCTCGGAAGCAAGCAGGTG
NDUFS1	Mus musculus	TCCAGTGTACCCGGTGCATC	AATGTATGTGCCAACTTGCATGTC
CD2AP	Mus musculus	TGTTTGTGACCACTAAGCTTGCTTC	TTAAGACCGGGCCCACTCTC
β-Tublin	Mus musculus	CAGCTGGACCGAATCTCTGTGTA	CCTGAGCGAACGGAGTCCATA
SLC7A11	Homo sapiens	CCCTATGCCAAACAGGTGAACA	GAAGACCCAATAAGTTTGCCGAAG
LRPPRC	Homo sapiens	CAAGGTGAGGCATAACGTTGG	AGTCACTTCATGCAAATGAGATGG
NDUFS1	Homo sapiens	TTGCCACGTATGCATGAGGA	TTTCTGACCATTGGCTCGGTAA
CD2AP	Homo sapiens	GAGGGAAGTTTCCAGCAGATTTCA	TCTGACTGAGGAGGACACAAGCA
β-Tublin	Homo sapiens	GGAACTCACCCAGCAGGTCT	TGCACGTTAAGCATCTGCTCA

### Construction of miRNA-mRNA-transcription factors (TFs) regulatory network

The miRNAs of the characterized genes were predicted using the miRNet database (https://www.mirnet.ca/) and the NetworkAnalyst database (https://www.networkanalyst.ca/). The miRNA-mRNA regulatory pairs were obtained by intersecting the results of the two databases. The mRNA-TF relationship pairs were obtained by transcription factor prediction analysis of the characterized genes using the NetworkAnalyst database (https://www.networkanalyst.ca/). The miRNAs and TFs regulated by the same mRNA were screened out, and the miRNA-mRNA-TF regulatory network was constructed using Cytoscape software^26^.

### Statistical analysis

All bioinformatics data were statistically analyzed using R 4.1.2. All experimental data were analyzed using SPSS 25.0 statistical software. All count data were expressed as percentages, measurement data were expressed as the mean ± standard deviation, and one-way ANOVA and the LSD-t test were used for measurement data analysis. In the text, *p < 0.05, **p < 0.01, ***p < 0.001 and ****p < 0.0001.

## Results

### Identification of differentially expressed DRGs and functional enrichment analysis

**Figure 2:**
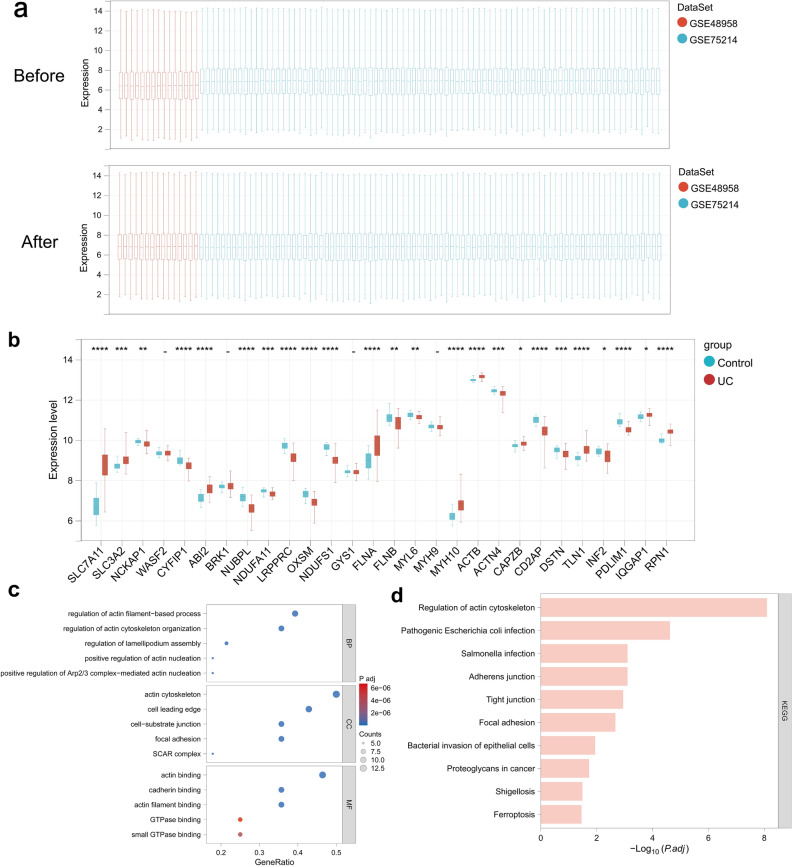
Expression and functional enrichment analysis of 28 DRGs in UC. (**a**) Batch treatment effect between the integrated datasets before and after debatching. (**b**) Box line plot showing the expression differences of DRGs in healthy individuals and UC patients. (**c**) Top five items of the differentially expressed DRGs GO functional enrichment analysis. (**d**) Top ten items of the differentially expressed DRGs KEGG functional enrichment analysis.

To investigate the expression of DRGs in the intestinal mucosa of UC patients, the GSE48958 and GSE75214 datasets were downloaded from the GEO database, and the training set containing 81 UC samples and 19 healthy control samples was obtained after debatch effect processing (Fig. 2a). The expression of DRGs in UC patients and healthy individuals in the training set was analyzed, and the results showed that a total of 24 DRGs were differentially expressed between the two groups. Compared with expression in healthy individuals, the expression of SLC7A11, SLC3A2, ABI2, FLNA, MYH10, TLN1, and RPN1 in the intestinal mucosa of UC patients was significantly elevated, while the expression of CYFIP1, NUBPL, NDUFA11, LRPPRC, OXSM, NDUFS1, etc., was decreased (Fig. 2b). GO and KEGG functional enrichment analyses of the differentially expressed DRGs showed that the above genes were mainly enriched for the regulation of actin filament-based processes and actin cytoskeleton organization, regulation of lamellipodium assembly (BP), cell leading edge, cell-substrate junction, focal adhesion (CC) and GTPase binding, small GTPase binding, actin binding, cadherin binding, actin filament binding (MF), and other pathways closely related to actin (Fig. 2c,d).

### Disulfide stress is present in the intestinal mucosal tissue of DSS-induced colitis mice and UC patients

**Figure 3:**
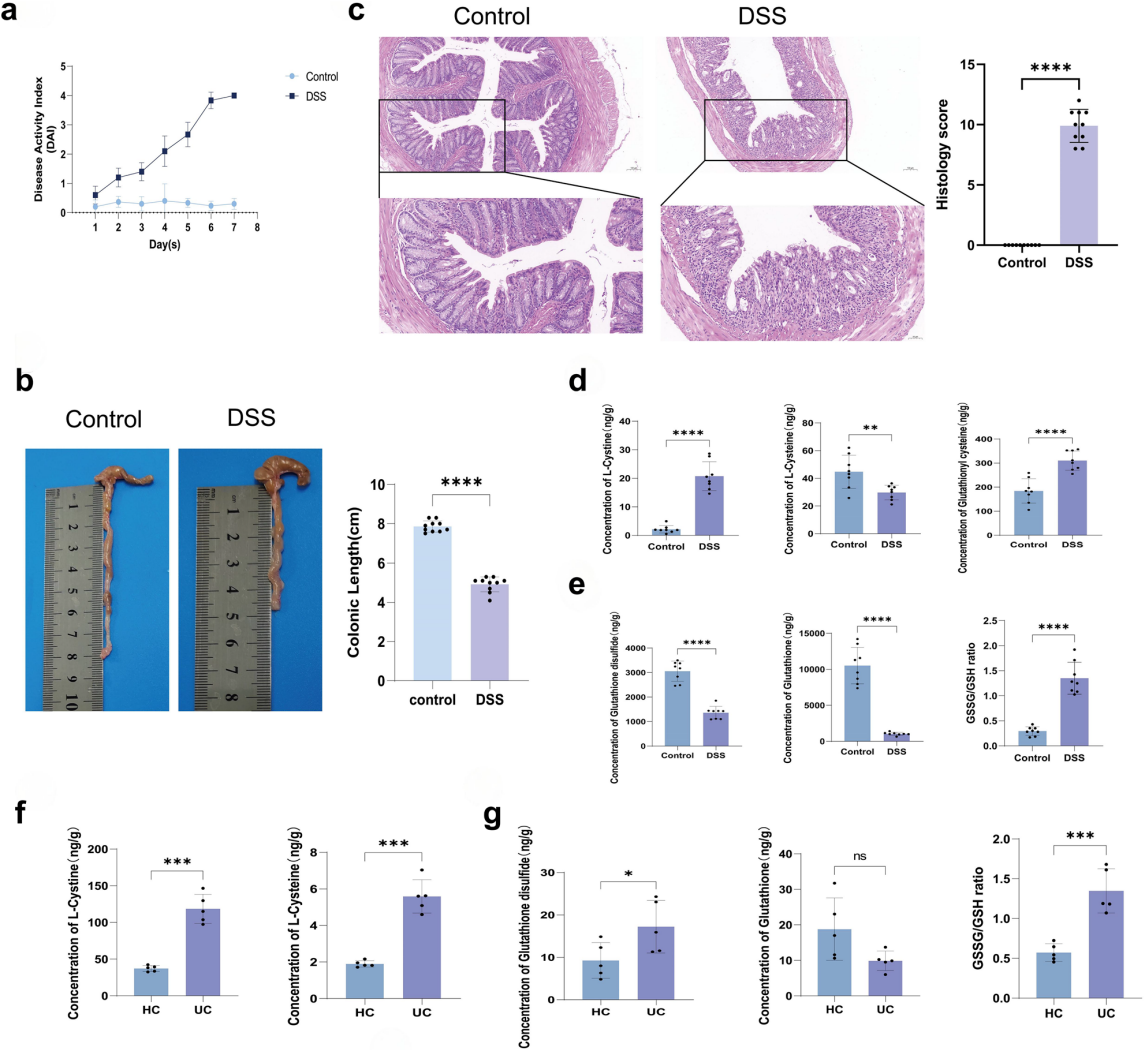
The presence of disulfide stress in intestinal mucosal tissues of DSS-induced colitis mice and UC patients. (**a**) Disease activity index scores of mice. (**b**) Representative pictures of the colon and statistics regarding its length in normal control and DSS-induced colitis mice. (**c**) HE-stained sections and histological scores of the colon of normal control and DSS-induced colitis mice. (**d**) The cystine (left), cysteine (middle), and glutathionyl-cysteine (right) contents in the intestinal tissues of normal control and DSS-induced colitis mice. (**e**) The GSSG (left) and GSH (middle) contents and GSSG to GSH ratio (right) of normal control and DSS-induced colitis mice. (**f**) The cystine and cysteine contents in intestinal tissues of healthy individuals and UC patients. (**g**) The GSSG (left)and GSH (middle) contents and the GSSG to GSH ratio (right) in intestinal tissues of healthy individuals and UC patients.

To clarify the presence of disulfide stress in UC, we constructed a mouse colitis model using a DSS solution at a concentration of 2.0 %. Compared with mice drinking distilled water, mice in the DSS group had significantly higher DAI scores (Fig. 3a) and a dramatic reduction in their colon length (Fig. 3b) after modeling. Microscopic observation of the intestinal mucosal tissue sections of mice revealed that the intestinal wall structure of control mice was intact and that the intestinal epithelial cells were neatly arranged. However, the intestinal wall structure of mice in the DSS group was hierarchically disorganized, with a significant loss of intestinal epithelial cells. A large number of inflammatory cells, such as lymphocytes and neutrophils, infiltrated the intestinal mucosa, and a significantly higher histological score was observed compared with that in the control group (Fig. 3c). The levels of cystine, cysteine, glutathione, and glutathione disulfide in the colonic tissues of UC patients and DSS mice were measured by HPLC–MS. The results showed that the levels of cystine and glutathionyl-cysteine in the intestinal mucosal tissues of DSS mice were significantly higher, the levels of cysteine were decreased (Fig. 3d), and the ratio of cystine/cysteine was significantly larger compared with that in control mice, suggesting the accumulation of disulfides in the intestinal mucosal tissues of DSS-induced colitis mice. Second, both the reduced glutathione (GSH) and glutathione disulfide (GSSG) contents were decreased in the DSS group of mice, but the decrease in GSH was more significant, and the ratio of GSSG/GSH was significantly increased compared with that in the drinking water group of mice (Fig. 3e). Similarly, the cystine content in the intestinal mucosal tissues of UC patients was significantly higher, and the cysteine content was also higher than that of healthy individuals, but the cystine/cysteine ratio was still higher than that of healthy individuals (Fig. 3f). In addition, the GSSG content was elevated and the GSH content was decreased in the intestinal mucosal tissues of UC patients, and the ratio of GSSG/GSH was significantly higher than that of healthy individuals (Fig. 3g). In summary, the intestinal mucosal tissues of DSS mice and UC patients may experience aberrant disulfide accumulation due to the inability of NADPH to meet the demand for reducing the increased cystine and GSSG levels to cysteine and GSH, respectively. That is, NADPH is in a state of relative deficiency and results in disulfide stress in DSS-induced colitis mice and UC patients, which suggests that UC is associated with disulfidptosis.

### Screening candidate genes closely related to UC based on DRGs

**Figure 4:**
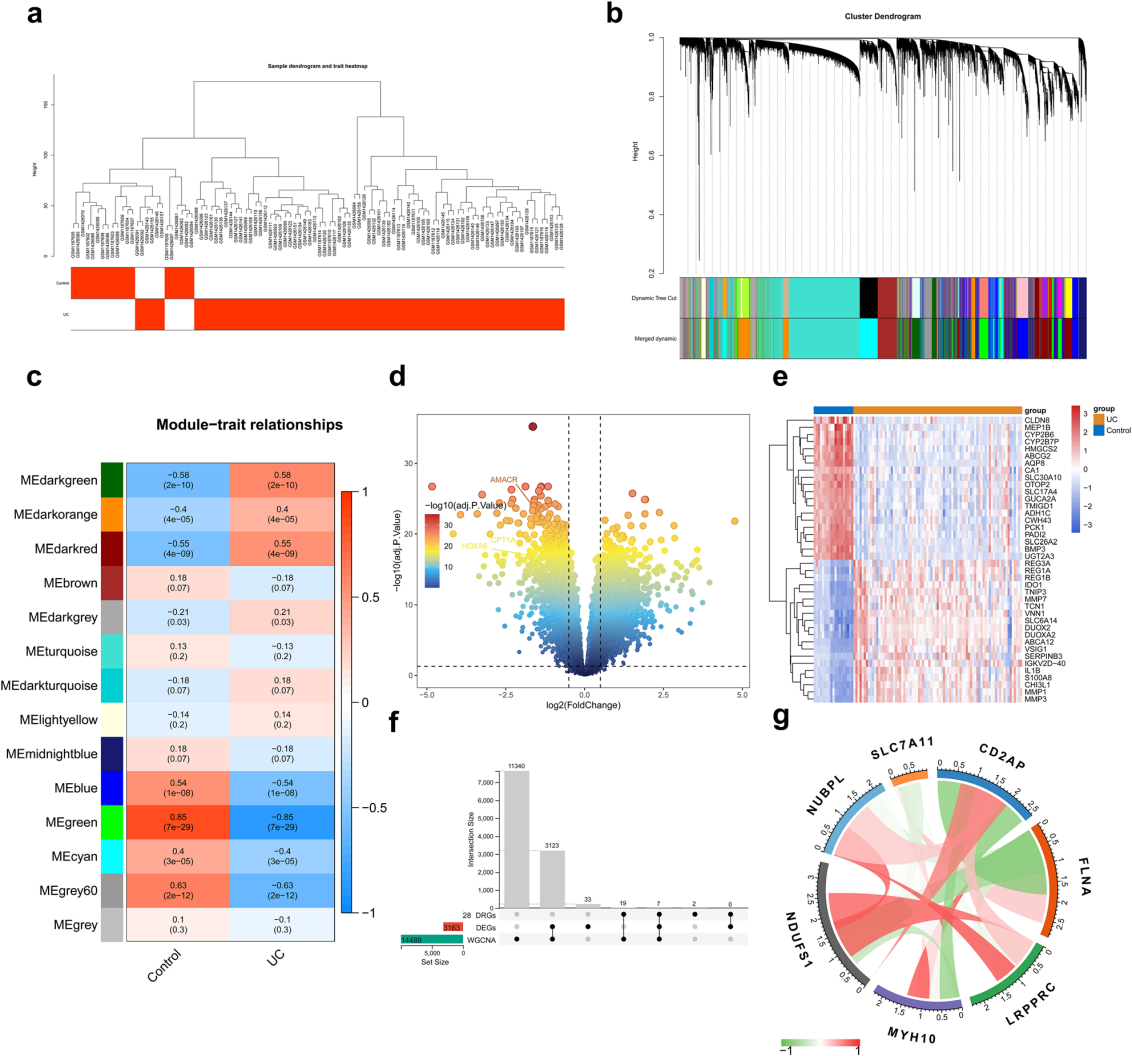
Screening candidate genes closely related to UC based on DRGs. (**a**) Clustering and phenotyping of samples in the dataset. Each branch in the figure represents a sample, the height in the vertical coordinates is the clustering distance, and the horizontal coordinates are the phenotypic traits to which the samples correspond. (**b**) Module clustering dendrogram. This graph indicates different genes in the horizontal direction and the uncorrelation between genes in the vertical direction; the lower the branch is, the less uncorrelation of the genes within the branch, i.e., the stronger the correlation. (**c**) Heatmap of the correlation between module trait genes and clinical traits. Vertical coordinates are different modules, horizontal coordinates are different traits, and each square indicates the correlation coefficient and significance p value for a module and a trait. (**d**) Volcano plot showing the DEGs between UC and healthy individuals. (**e**) Heatmap showing the TOP 20 upregulated and downregulated DEGs between UC and healthy individuals. (**f**) UpSet plot showing the number of candidate genes obtained from the intersection of the DEGs, hub genes, and the 28 DRGs. (**g**) Circos plot showing the number of genes and correlations between candidate genes.

To explore the key DRGs involved in UC, we used UC and healthy controls as traits and performed WGCNA on all genes to search for modular genes that are highly correlated with the UC phenotype. All the samples in the training set were clustered, and the results showed that the samples were well clustered and that no abnormal samples needed to be eliminated (Fig. 4a). The gene module clustering dendrogram was obtained by calculating the similarity between genes, determining a soft threshold of 8, setting MEDissThres to 0.3, and obtaining 14 modules by merging the dynamic shear tree algorithm aggregation (Fig. 4b). We identified the most relevant modules (*p* value < 0.05 and correlation coefficient of 0.4) with UC and healthy controls as key modules, which included dark red, green, blue, cyan, dark green, dark orange, and gray, for a total of seven modules (Fig. 4c). A total of 14,489 hub genes were obtained by merging the key modules (Supplementary Table S5).

Next, we analyzed the differentially expressed genes between UC and healthy controls and found that there were 3163 genes with significant differences in expression. Compared with healthy controls, 1747 genes were upregulated and 1416 genes were downregulated in UC colon tissues, as shown in the volcano diagram (Fig. 4d,e; Supplementary Table S6). Seven candidate genes were obtained by taking the intersection of the differentially expressed genes, the hub genes, and the 28 DRGs (Fig. 4f; Supplementary Table S7). We further analyzed the correlations between the candidate genes, and the results showed that most of the candidate genes were highly correlated with each other, except for SLC7A11, wherein there was a strong significantly negative correlation between FLNA and CD2AP and a dramatically strong positive correlation between NUBPL and LRPPRC (Fig. 4g; Supplementary Table S8).

### Machine learning screening for the characterized genes

**Figure 5:**
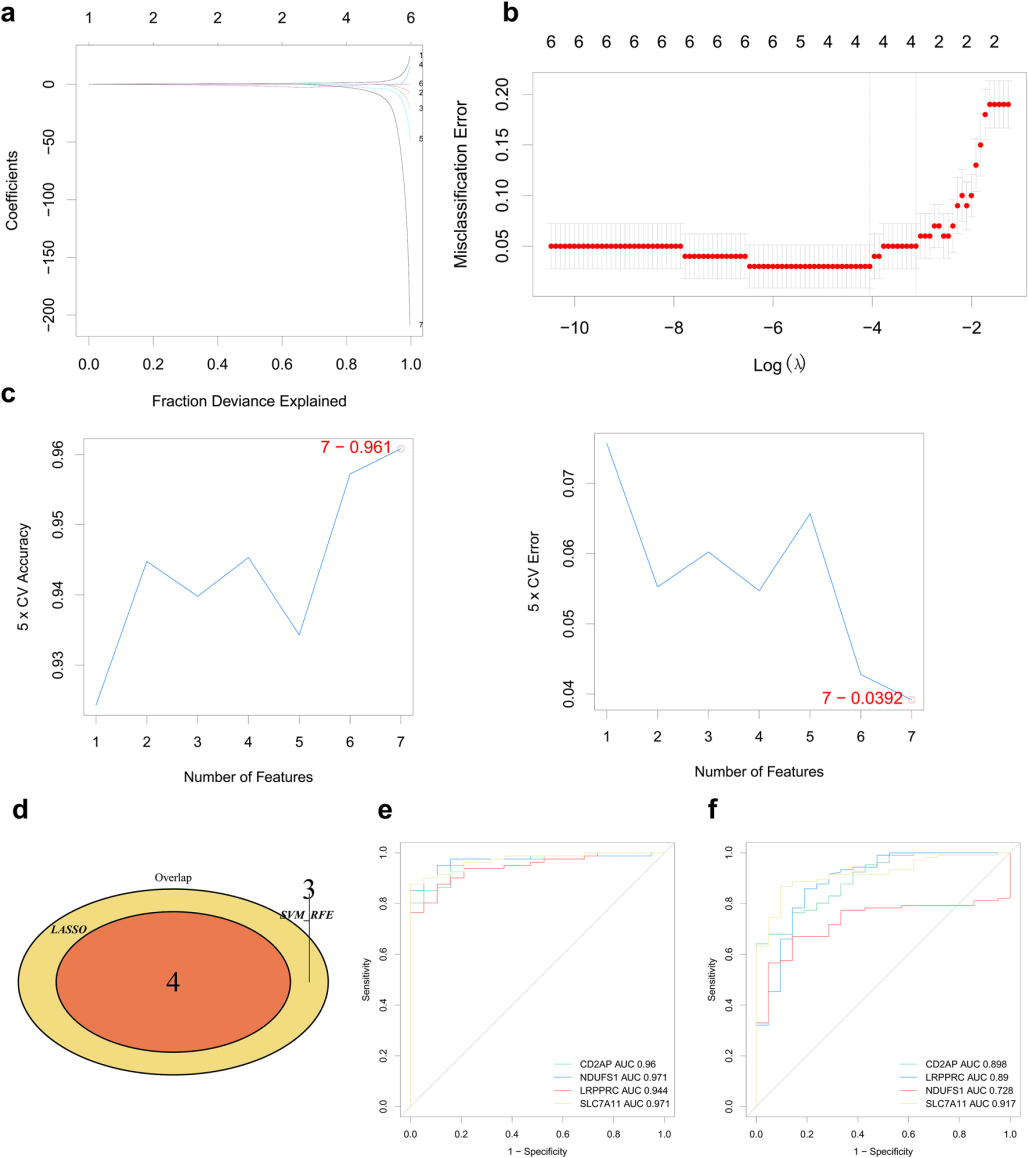
Machine learning screening for the characterized genes. (**a**,**b**) Constructing the LASSO logistic regression analysis for screening the characterized genes. (**a**) Plot of the gene coefficient changes. The LASSO coefficient path plots of the seven candidate genes are displayed. Each curve represents the trajectory of each hub gene, the vertical coordinate is the value of the gene, the lower horizontal coordinate is log (λ), and the upper horizontal coordinate is the number of nonzero hub genes in the model at this time. (**b**) LASSO regression cross-validation curves using tenfold cross-validation to determine the optimal λ value. (**c**) Support vector machine analysis for screening the characterized genes. Type accuracy (left) vs. error rate (right) using fivefold cross-validation of different feature combination models. (**d**) Venn diagram showing the intersection of the characterized genes obtained by the two algorithms, LASSO logistic regression and SVM-RFE. (**e**) ROC curves analyzing the diagnostic value of the characterized genes in the training set. (**f**) ROC curves verifying the diagnostic value of the characterized genes in the validation set.

We used LASSO and SVM algorithms to screen the UC disulfidptosis signature genes from the above seven candidate genes. Based on the LASSO logistic regression analysis, four feature genes were identified, namely, SLC7A11, LRPPRC, NDUFS1, and CD2AP (Fig. 5a,b; Supplementary Table S9). Second, the SVM algorithm was used to screen and obtain a total of seven feature genes, included CD2AP, SLC7A11, FLNA, NUBPL, LRPPRC, MYH10, and NDUFS1 (Fig. 5c; Supplementary Table S10). The intersection of the two algorithms was taken to obtain four feature genes, SLC7A11, LRPPRC, NDUFS1, and CD2AP (Fig. 5d). To evaluate the diagnostic value of each feature gene for UC, the sensitivity and specificity of each feature gene in the training set were analyzed by ROC curves. The results showed that the AUC values of the four feature genes reached 0.9 or more, which indicates that the above feature genes have the diagnostic ability to differentiate between the disease and control samples (Fig. 5e; Supplementary Table S11). We performed ROC curve analysis in the GSE87473 validation set to verify the diagnostic ability of each feature gene. The results showed that the AUC value of each feature gene in the validation set reached over 0.7, which further validated the diagnostic value of the feature genes (Fig. 5f; Supplementary Table S12).

### Functional enrichment analysis of the characterized genes

**Figure 6:**
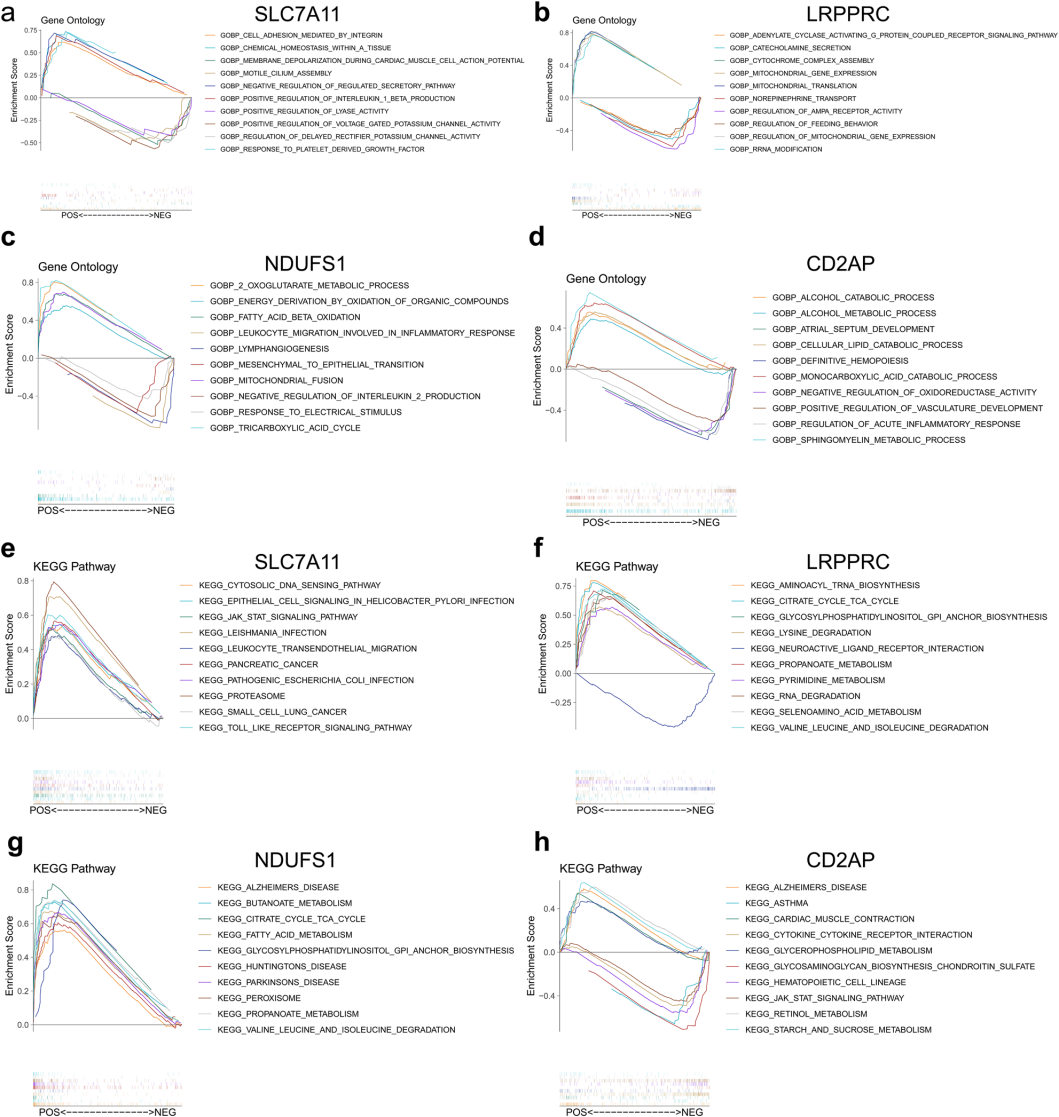
GSEA functional enrichment analysis of the characterized genes. (**a**–**d**) Top ten results of the functional enrichment analysis of the characterized genes in GO BP. (**e**,**h**) Top ten results of the KEGG pathway enrichment analysis of the characterized genes.

To investigate the possible functions of SLC7A11, LRPPRC, NDUFS1, and CD2AP in UC, we performed GSEA functional enrichment analysis of these genes. The GO-BP results showed that SLC7A11 was mainly positively correlated with cell adhesion mediated by integrin, chemical homeostasis within a tissue, negative regulation of regulated secretory pathway, positive regulation of interleukin 1 beta production, etc. (Fig. 6a). LRPPRC was mainly enriched in mitochondrial translation, mitochondrial gene expression, regulation of mitochondrial gene expression, etc. (Fig. 6b). NDUFS1 and CD2AP were enriched in metabolism-related pathways. NDUFS1 was mainly positively correlated with the 2-oxoglutarate metabolic process, energy derivation by oxidation of organic compounds, fatty acid beta-oxidation, and was negatively correlated with leukocyte migration involved in the inflammatory response, negative regulation of interleukin 2 production, etc. (Fig. 6c). CD2AP was mainly positively associated with the alcohol catabolic process, alcohol metabolic process, sphingomyelin metabolic process, cellular lipid catabolic process, and monocarboxylic acid catabolic process, which were negatively correlated with with positive regulation of vasculature development, regulation of acute inflammatory response, atrial septum development, negative regulation of oxidoreductase activity, and definitive hematopoiesis (Fig. 6d). KEGG analysis showed that SLC7A11 was positively correlated with the Toll-like receptor signaling pathway, leukocyte transendothelial migration, and JAK-STAT signaling pathway (Fig. 6e). Both LRPPRC and NDUFS1 were significantly enriched in the citrate cycle, TCA cycle, valine, leucine and isoleucine degradation, propanoate metabolism pathways, etc. (Fig. 6f,g). CD2AP was positively correlated with the metabolism of sugar and glycerol phospholipids and negatively correlated with cytokine-cytokine receptor interactions and the JAK-STAT signaling pathway (Fig. 6h).

### Evaluation of immune infiltration in UC

**Figure 7:**
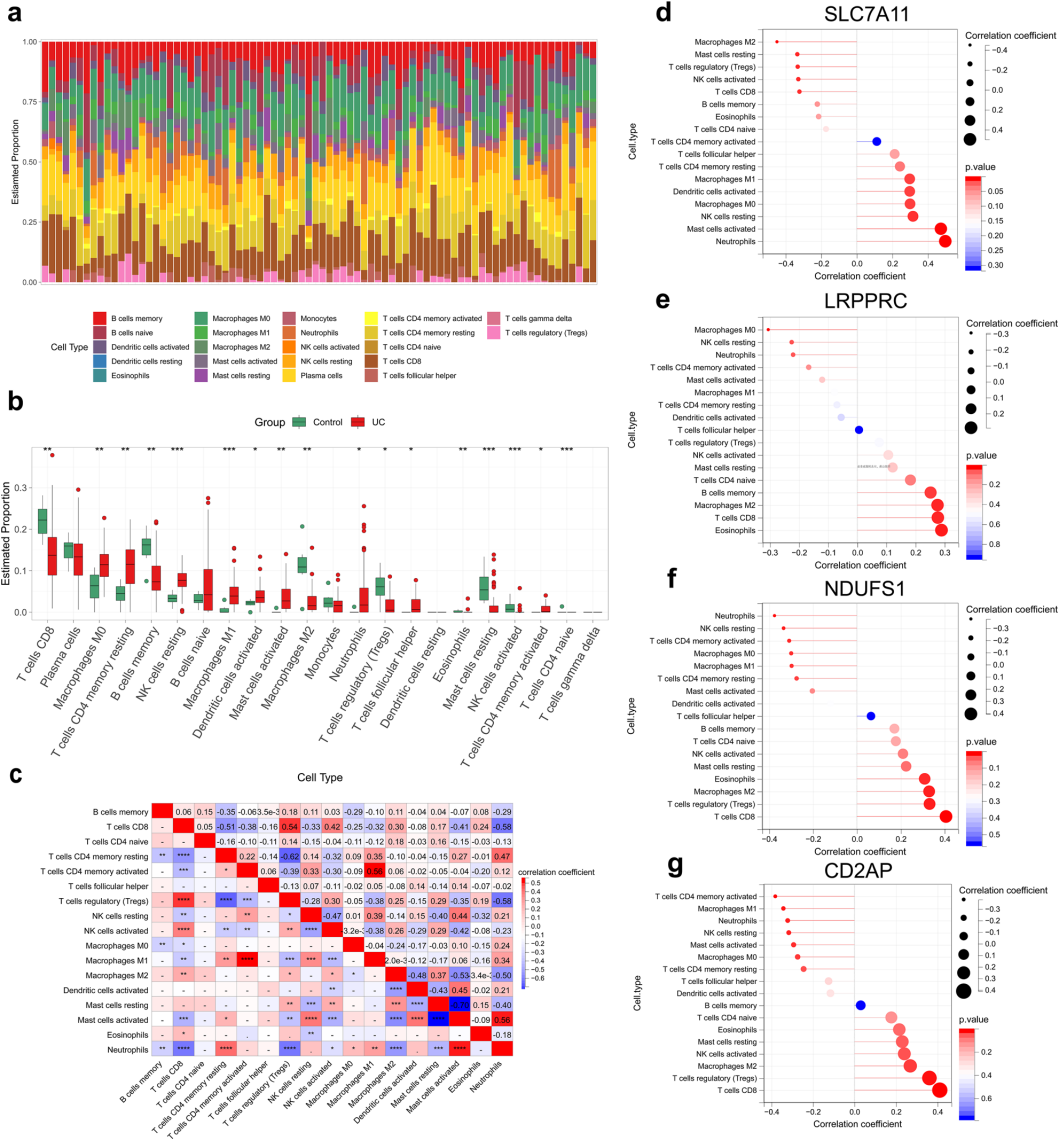
Evaluation of immune cell infiltration in UC. (**a**) Immune cell scoring plots of the training set samples showing the difference in infiltration abundance of 22 immune cells among healthy individuals and UC patients. (**b**) Box line plots showing the infiltration abundance of 22 immune cells in healthy individuals and UC patients. (**c**) Heatmap showing the correlations between the differentially abundant immune cells. (**d**–**g**) Lollipop plots showing the correlations of the characterized genes with the differentially abundant immune cells.

The abundance of 22 immune cells was shown in the heat map (Fig. 7a; Supplementary Table S13). Seventeen immune cells were significantly different between the UC samples and the control samples (*p* < 0.05) (Fig. 7b). M0 macrophages, resting memory CD4 + T cells, resting NK cells, M1 macrophages, and activated dendritic cells were significantly more infiltrated in UC. In contrast, the abundance of immune cells, such as CD8 + T cells, memory B cells, M2 macrophages, and regulatory T cells , was lower in UC patients than in healthy individuals. The results of the correlations among the differentially abundant immune cells showed that most of the differentially abundant immune cells were correlated with each other, except CD4 + naive T cells and follicular helper T cells. Activated memory CD4 + T cells had the strongest positive correlation with M1 macrophages, activated mast cells were strongly positively correlated with neutrophils, and resting mast cells had the strongest negative correlation with activated mast cells (Fig. 7c; Supplementary Table S14). We further analyzed the correlations among the characterized genes and the differentially abundant immune cells, and the results showed that SLC7A11 had the strongest positive correlation with neutrophils and the strongest negative correlation with M2 macrophages (Fig. 7d–g; Supplementary Table S15).

### Expression of the characterized genes in the intestinal mucosa of DSS-induced colitis mice and UC patients

**Figure 8:**
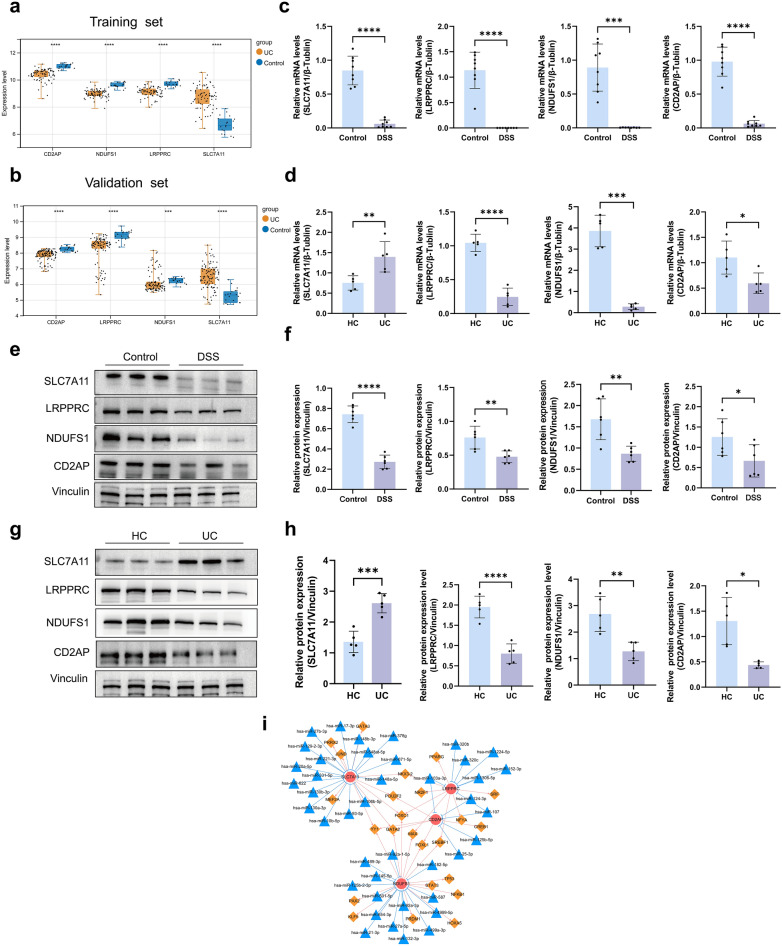
Expression of the characterized genes in the intestinal mucosa of DSS-induced colitis mice and UC patients and the miRNA-mRNA-TF network. (**a**) Differences in the expression of feature genes in UC and healthy individuals were analyzed in the training set. (**b**) Validation of the expression differences in the characterized genes in UC and healthy individuals in the validation set. (**c**) The qRT-PCR to detect the mRNA expression levels of the characterized genes in the intestinal mucosa of normal control and DSS-induced colitis mice. (**d**) The qRT-PCR detection of the mRNA expression levels of the characterized genes in the intestinal mucosa of healthy individuals and UC patients. (**e**,**f**) Western blotting to detect the protein expression levels of the characterized genes in the intestinal mucosa of normal control and DSS-induced colitis mice. (**g**,**h**) Western blotting to detect the protein expression levels of the characterized genes in the intestinal mucosa of healthy people and UC patients. (The samples derive from the same experiment and that blots were processed in parallel. We cropped the membranes according to the molecular weight of the target proteins, resulting in the absence of some images of adequate length. Therefore, we uploaded the original images of all target proteins for human and animal samples in the supplementary material.) (**i**) The miRNA-mRNA-TF regulatory network diagram (red circles indicate the characterized genes, yellow diamonds indicate TFs, and blue triangles indicate miRNAs).

To clarify the expression levels of the four characterized genes in the intestinal mucosa of UC patients, we analyzed their expression levels in the training set and validated them in the validation set. The results showed that the expression of SLC7A11 was upregulated and that the expression of CD2AP, NDUFS1, and LRPPRC was significantly downregulated in UC patients compared with healthy controls (Fig. 8a,b). We further examined the mRNA expression levels and protein expression levels of the characterized genes by qRT-PCR and western blotting, respectively, in the intestinal mucosal tissues of DSS-induced colitis mice and UC patients, and the results showed that the mRNA expression levels and protein levels of SLC7A11, CD2AP, NDUFS1, and LRPPRC in DSS mice were significantly decreased (Fig. 8c,e,f; Supplementary Fig. S1). The mRNA expression levels and protein expression levels of SLC7A11 were significantly increased, and the mRNA expression levels and protein levels of CD2AP, NDUFS1, and LRPPRC were significantly decreased in UC patients (Fig. 8d,g,h; Supplementary Fig. S2).

To understand the potential regulatory mechanisms of the characterized genes in UC, we used the miRNet database and NetworkAnalyst database to analyze the miRNA and TF prediction of the feature genes and to construct miRNA-mRNA-TF regulatory networks, and a total of 416 miRNA-mRNA-TF relationships were obtained, which contained 41 miRNAs, 4 mRNAs and 24 TFs (Fig. 8i; Supplementary Table S16).

## Discussion

The unclear pathogenesis is the critical reason for the poor treatment response of UC. Imbalanced programmed cell death of intestinal mucosal cells is one of the important factors facilitating the development of UC^2^. Disulfidptosis is a recently identified type of programmed cell death, and in this study, its link to the pathogenesis of UC was assessed through the examination of the expression levels of DRGs in the intestinal mucosa of UC and the analysis of the accumulation of disulfides, such as cystine, in the intestinal mucosa to identify key DRGs that may play a role in UC.

To confirm whether UC is associated with disulfidptosis, we analyzed the expression levels of 28 DRGs in UC intestinal mucosal tissues by utilizing public databases. The results showed that most of the DRGs were differentially expressed in healthy individuals and patients with UC. Among these genes, those that promote disulfidptosis, such as SLC7A11, SLC3A2, and RPN1, were highly expressed in UC. Genes that inhibit disulfidptosis, such as NUBPL, NDUFA11, LRPPRC, OXSM, and NDUFS1, were significantly downregulated in UC. In summary, the results show that disulfidptosis may exist in UC and that the related genes might play important roles in UC. We performed GO and KEGG functional enrichment analyses of differential DRGs, and the results showed significant enrichment in the pathways of actin assembly, regulation, and binding. The study also demonstrated that in cells undergoing disulfidptosis, there is increased disulfide bond formation in actin skeleton proteins, leading to the contraction of actin filaments and cytoskeletal disruption, resulting in cell death^9^.

Disulfide stress caused by disulfide accumulation is an important prerequisite for inducing cells to undergo disulfidptosis. It was shown that disulfidptosis could be caused by the aberrant accumulation of disulfides in cells with high SLC7A11 expression^9,27^. Cystine transport is mediated by the cystine/glutamate reverse transport system composed of SLC7A11 and SLC3A2^11^. Our results showed that the expression levels of SLC7A11 and SLC3A2 were significantly elevated in UC patients compared to healthy individuals. Targeted metabolomics by HPLC‒MS on intestinal mucosal tissues from DSS-induced colitis mice and UC patients was performed to clarify the changes in cystine. The results showed that the levels of disulfides (cystine and glutathione-cysteine) in the intestinal mucosal tissues of DSS mice were significantly higher than those of control mice. Additionally, the levels of cysteine were decreased, and the ratio of cystine/cysteine was significantly larger than that of control mice. These findings suggest disulfide accumulation in the intestinal mucosal tissues of DSS mice. Under normal conditions, cystine is transported from the extracellular space to the intracellular space by SLC7A11 and is reduced to cysteine by NADPH^27^. Higher SLC7A11 expression in the colon of DSS mice indicated that more cystine was transported intracellularly. However, the amount of cysteine in the DSS group of mice was decreased. This indicates that the increased cystine failed to be reduced to cysteine by enough NADPH, suggesting that there is an accumulation of cystine in the DSS mice. Normally, GSH is produced by glutathione reductase using NADPH to catalyze GSSG, and GSH can be regenerated using the NADPH cycle after GSH depletion^28^. However, our results showed that both GSH and GSSG levels were decreased in the DSS group of mice, but the decrease in GSH was more pronounced. Additionally, the ratio of GSSG/GSH was significantly higher compared with that in the drinking water group, suggesting that NADPH may not be able to satisfy the demand for GSH replenishment by GSSG reduction in the current disease state. This also means that NADPH is relatively lacking in the DSS mouse colon. Similarly, the cystine content was significantly elevated in the intestinal mucosal tissues of UC patients compared with healthy individuals, which is consistent with previous findings^15^. In addition, cysteine levels were also higher in UC patients than in healthy individuals, but the cystine/cysteine ratio was still higher than that in healthy individuals. The elevated levels of GSSG and decreased levels of GSH in the intestinal mucosal tissues of patients with UC, with a significantly higher GSSG/GSH ratio than that in healthy individuals, suggest that there is also an abnormal accumulation of cystine, GSSG, and other disulfide compounds in the intestinal mucosa of UC and that there is a relative deficiency of NADPH that could not address the need for reduction of increased cystine and GSSG to cysteine and GSH, respectively. In summary, the presence of the abnormal accumulation of disulfides in the intestinal mucosal tissues of DSS mice and UC patients and the relative deficiency of NADPH suggest that disulfide stress occurs in DSS-induced colitis mice and UC patients.

We jointly screened seven DRGs associated with UC by differential analysis and WGCNA and further used machine learning to obtain four characterized genes, SLC7A11, NDUFS1, LRPPRC, and CD2AP. ROC curve analysis showed that the characterized genes have the potential to diagnose UC. We further tested the mRNA and protein levels of the characterized genes in the intestinal mucosa of DSS-induced colitis mice and UC patients, and the results showed that the mRNA and protein levels of SLC7A11, NDUFS1, LRPPRC, and CD2AP were dramatically downregulated in DSS-induced colitis mice. Compared with those in healthy individuals, the expression levels of SLC7A11 were upregulated and those of NDUFS1, LRPPRC, and CD2AP were downregulated in UC patients, consistent with the mRNA expression levels. The different modes and mechanisms of SLC7A11 involved in the regulation of disulfidptosis may exist in the intestinal mucosa of patients with UC versus those of DSS-induced colitis mice and should be further explored. SLC7A11 is a cystine/glutamate reverse transport protein that can inhibit cellular oxidative stress and maintain cellular redox balance to arrest cell death caused by lipid peroxidation^29,30^. Some studies have reported that high-fat diets can ameliorate DSS-induced colitis by inhibiting iron death through upregulation of SLC7A11^31^. However, recent studies have shown that under glucose starvation conditions, the abnormal accumulation of disulfides, such as cystine, occurs in cells with high SLC7A11 expression, causing disulfide stress, which induces rapid cell death^9^. Based on the dataset and our experimental results, the expression of SLC7A11 was upregulated and disulfide stress was present in UC intestinal mucosa. These findings demonstrate that UC is associated with disulfidptosis, but the specific mechanism of action needs to be further explored. LRPPRC encodes a leucine-rich pentatricopeptide repeat protein that is mainly localized in the mitochondrion and regulates the maturation, stability, and cellular oxidative phosphorylation of mitochondrial mRNA (mt-mRNA)^32,33^. LRPPRC serves as a key posttranscriptional regulator of mitochondrial DNA expression and is required for polyadenylation and coordination of mt-mRNA translation^34^. NDUFS1 encodes the largest core subunit of mitochondrial respiratory chain complex I, regulates the formation of mitochondrial reactive oxygen species (ROS) and plays an important role in controlling respiratory chain complex I activity^35–39^. Upregulation of NDUFS1 improves mitochondrial respiratory function by attenuating mitochondrial dysfunction and enhancing respiratory chain complex I activity and reduces oxidative stress and apoptosis induced by cardiac ischemia^40^. Studies have shown that LRPPRC and NDUFS1 are resistant to disulfidptosis, and their inactivation synergizes with glucose starvation to induce cell death^9^. LRPPRC and NDUFS1 are distinctively downregulated in UC, suggesting that they may play important synergistic roles in inducing disulfidptosis in UC. CD2AP encodes the cytoskeletal protein CD2-associated protein, which plays a pivotal role in regulating the assembly of the cytoskeleton and intercellular adhesion and is involved in receptor-mediated endocytosis^41,42^. Studies have shown that CD2AP-deficient mice have a significantly lower number of podocytes, which correlates with the upregulation of podocyte apoptotic pathways^43^. In conclusion, SLC7A11, NDUFS1, LRPPRC, and CD2AP can all participate in the regulation of cell survival, but their specific roles and mechanisms in UC have not been reported and need to be further explored.

To explore the possible functions of SLC7A11, NDUFS1, LRPPRC, and CD2AP in UC, we performed GSEA. SLC7A11 was predominantly enriched in the positive regulation of the interleukin 1 beta production pathway, and many studies have shown that interleukin 1 beta (IL-1β), which is elevated in DSS-induced colitis mice and UC, acts as a proinflammatory factor mediating the disruption of intestinal barrier function^44^. Cell adhesion mediated by integrin is also one of the major pathways in which SLC7A11 is enriched. Studies have shown that the intestinal migration of lymphocytes is an important factor causing an abnormal immune response in UC^45^. Integrin is a key mediator of the intestinal migration of lymphocytes. It mediates the stable adhesion of lymphocytes to endothelial cells by binding to adhesion molecules expressed on the surface of the vascular endothelium^46^. This suggests that the elevation of SLC7A11 levels in UC intestinal mucosal tissues may participate in the development of UC by promoting IL-1β secretion and the intestinal migration of lymphocytes. LRPPRC was significantly enriched in pathways related to mitochondrial translation, mitochondrial gene expression and regulation. Mitochondria play a crucial role in a variety of cell physiological processes, including cell proliferation, differentiation, energy production, and programmed cell death^47,48^. Mitochondria are an important synthetic factory for NADPH^49^, a key substance in the regulation of disulfide stress, and thus, the functional status of mitochondria may be closely related to the occurrence and development of disulfidptosis. LRPPRC deficiency reduces mitochondrial mRNA stability, leading to loss of mitochondrial mRNA polyadenylation and aberrant mitochondrial translation^33,34^. In cardiac mitochondria lacking LRPPRC, respiratory chain complex IV undergoes severe defects, and the homeostasis of its cytochrome C oxidase subunit I (COX I) and cytochrome C oxidase subunit II (COX II) is significantly reduced, leading to mitochondrial dysfunction^34^. Additionally, NDUFS1 was enriched in the mitochondrial fusion pathway. Mitochondrial fusion is a cytosolic endogenous protective program for mitochondrial quality monitoring that is essential for maintaining mitochondrial homeostasis and function^50,51^. NDUFS1 encodes the largest core subunit in respiratory chain complex I. When NDUFS1 expression is downregulated, the level of this subunit is decreased, leading to reduced homeostasis and mitochondrial dysfunction in respiratory chain complex I^37^. This study shows that LRPPRC and NDUFS1 levels are significantly decreased in UC, which may disrupt the homeostasis of the relevant subunits of respiratory chain complex IV and respiratory chain complex I. This, in turn, leads to the dysfunction of the respiratory electron transport chain and ultimately inhibits the production of NADPH and disulfide reduction, thus promoting the occurrence of disulfidptosis^52^.

In addition, NDUFS1 was positively correlated with pathways such as the 2-hydroxyglutarate metabolic process and TCA cycling pathway. Studies have reported that an increase in 2-hydroxyglutarate can lead to a decline in the number and impaired function of Th17 cells and inhibit the expression of interleukin 17A (IL-17A)^53^. More research evidence supports that IL-17A in the intestinal mucosa of patients with IBD may have a protective role against intestinal inflammation^54^. It is suggested that NDUFS1 may inhibit intestinal inflammation by promoting 2-hydroxyglutarate metabolism, weakening its inhibitory effect on IL-17A expression and promoting IL-17A secretion. NDUFS1 is downregulated in UC. 2-Hydroxyglutarate metabolism is decreased and accumulated. IL-17A secretion is reduced and fails to alleviate intestinal inflammation. The tricarboxylic acid (TCA) cycle serves as the body's primary mode of energy acquisition, and the TCA cycle is affected to some extent by the state of intestinal inflammation. It was found that the levels of intermediate metabolites of the tricarboxylic acid cycle, citric acid, transbutenedioic acid, isocitric acid, malic acid, and succinic acid were significantly decreased in the colon of UC patients^55^, and the levels of transbutenedioic acid were significantly elevated in the serum of UC patients^56^. There were significant differences in the pathways involved in the TCA cycle between UC patients and healthy individuals^57^. The expression of NDUFS1 was downregulated in UC, which may give rise to the decreases in the TCA cycle, and the levels of its intermediate metabolites would also decrease. CD2AP was positively correlated with the cellular lipid catabolic process pathway. Abnormal lipid metabolism is one of the characteristics of UC^58^, and proinflammatory factors in inflammation can inhibit the expression of lipoprotein lipase (LPL), which leads to a decrease in the levels of low-density lipoprotein cholesterol (LDL-C), high-density lipoprotein cholesterol (HDL-C), and very-low-density lipoprotein (VLDL) unsaturated lipids^59,60^. The expression of CD2AP is downregulated in UC, which may contribute to the level of lipid catabolic processes, and the levels of metabolites, such as LDL-C, will decrease. The above study suggests that UC disulfidptosis-related signature genes may be involved in the disease process of UC by regulating the body's metabolic and immune response.

**Figure 9:**
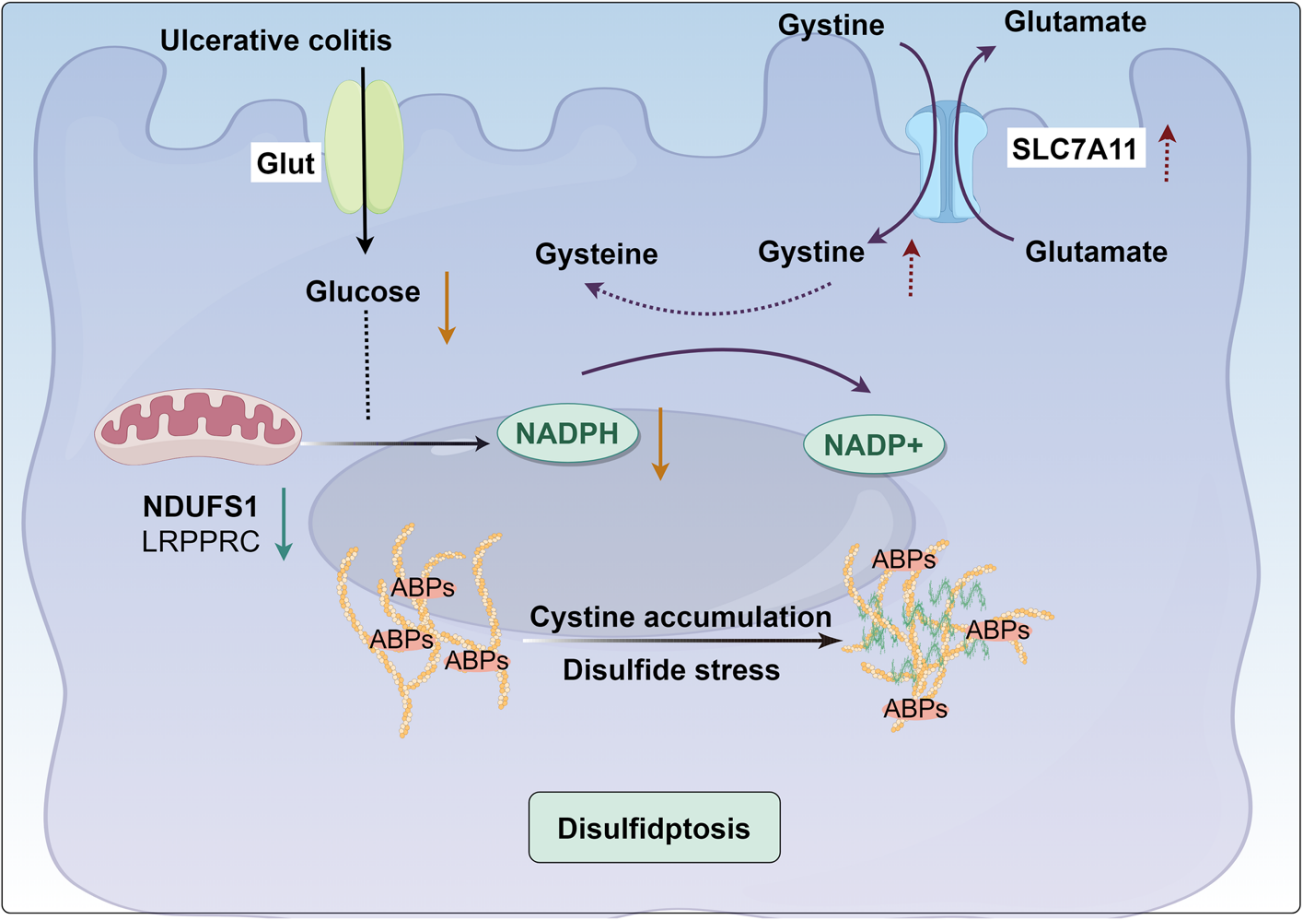
Potential mechanisms of UC disulfidptosis. In UC intestinal mucosa, the expression of SLC7A11 is elevated and its mediated cystine entry into the cell is significantly increased. At the same time, glucose deficiency caused by increased consumption and impaired absorption and mitochondrial dysfunction triggered by decreased expression of NDUFS1 and LRPPRC can lead to a decrease in NADPH synthesis, which is insufficient to completely reduce the excess cystine, and the large amount of accumulated cystine will trigger disulfide stress, leading to the increase in the formation of disulfide bonds between actin backbone proteins, and ultimately contributing to the intestinal mucosal disulfidptosis. In the figure, red dotted arrow showed the expression level of SLC7A11 was upregulated and cytine was increased in the UC intestinal mucosa. Orange solid arrows indicated that the glucose and NADPH was reduced, which were in relative deficiency in the intestinal mucosa of UC patients. Green solid arrow showed the expression level of NDUFS1 and LRPPRC were downregulated.

The aberrant immune response is an important cause of UC, and abnormal infiltration of multiple immune cells is a typical pathological feature^61^. We evaluated immune cell infiltration in UC and screened a total of 17 differential immune cells with different abundances in UC patients and healthy individuals. Correlations between the characterized genes and immune cells showed that the strongest positive correlation existed between SLC7A11 and neutrophils, and the strongest negative correlation existed between SLC7A11 and M2 macrophages. Neutrophils produce a variety of inflammatory cytokines, such as tumor necrosis factor alpha (TNF-α), interleukin-1 beta (IL-1β), and interleukin 6 (IL-6), which are involved in the regulation of innate and adaptive immune responses and are highly associated with intestinal mucosal inflammation. Currently, neutrophil infiltration is regarded as a hallmark of UC, and the degree of infiltration correlates with the disease severity of UC. Recent meta-analyses have shown that neutrophil infiltration is associated with the risk of UC recurrence and is the only independent predictor of the ability to maintain endoscopic and histologic improvement after biologics-induced therapy^62^. M2 macrophages are an important subtype of macrophages that produce anti-inflammatory factors, such as interleukin 10 (IL-10), tumor necrosis factor β (TGF-β)^63^, and can promote the proliferation and differentiation of Treg cells through IL-10 to play an anti-inflammatory role^64^. Additionally, it downregulates the levels of the proinflammatory factors IL-6 and inducible nitric oxide synthase (iNOS) to alleviate intestinal inflammation^65^. In addition, it promotes phagocytic activity to phagocytose apoptotic intestinal epithelial cells as well as pathogenic bacteria^66^. Studies have shown that M2 macrophages can secrete prostaglandin E2 (PGE2) and WNTs to promote the proliferation and differentiation of intestinal stem cells, in turn maintaining the integrity of the intestinal barrier and promoting the healing of the intestinal mucosa^67^. The expression of SLC7A11 was significantly upregulated in UC, suggesting that it may be involved in the development of UC by promoting the migration or infiltration of centrocytes and inhibiting the polarization or function of M2 macrophages.

In this study, we explored the relationship between disulfidptosis and UC and preliminarily explored its role and potential regulatory mechanisms in UC. However, there are still some limitations. The sample size for experimental validation was small, and some of the data in the study were obtained from public databases with limited sample information in the dataset, which could potentially bias the results. In addition, the regulation of disulfidptosis may involve intracellular actin cytoskeletal proteins and a variety of other proteins. In the future, we will further study the other pathways that may play a role in disulfidptosis and explore the regulatory mechanisms of disulfidptosis in UC.

## Conclusions

As a newly discovered mode of programmed cell death, disulfidptosis may play an important role in the regulation of intestinal mucosal homeostasis in UC. The new UC signature genes screened on the basis of disulfidptosis may provide a new theoretical basis for the further elucidation of the role and mechanisms of disulfidptosis in UC, as well as for exploring new pathogenic mechanisms and potential therapeutic targets of UC **(**Fig. 9**)**.

### Supplementary Information

Supplementary Information 1.

Supplementary Information 2.

Supplementary Information 3.

Supplementary Information 4.

Supplementary Information 5.

Supplementary Information 6.

Supplementary Information 7.

## Data Availability

The datasets presented in this study can be found in online repositories. The original contributions presented in the study can be found in online repositories (https://ngdc.cncb.ac.cn/omix/release/OMIX005126, https://ngdc.cncb.ac.cn/omix/release/OMIX005124) and in this manuscript and its Supplementary Information file. Further inquiries can be directed to the corresponding author.
