# Supplementary material for: Comprehensive analysis of disulfidptosis-related genes reveals the effect of disulfidptosis in ulcerative colitis
Source: Sci Rep. 2024 Jul 8;14:15705. doi: 10.1038/s41598-024-66533-9 (PMC11231342; doi:10.1038/s41598-024-66533-9)
Supplement: Supplementary file 1 — Supplementary Information 1. [file 41598_2024_66533_MOESM1_ESM.pdf]

Supplementary Information

Comprehensive analysis of disulfidptosis-related genes reveals the effect of disulfidptosis in ulcerative colitis

Huixian Song, Fengrui Zhang, Xinyu Bai, Hao Liang, Junkun Niu, Yinglei Miao

Corresponding author: Yinglei Miao. Department of Gastroenterology, The First Affiliated Hospital of Kunming Medical, Kunming , Yunnan Province, China. E-mail: miaoyinglei@yeah.net

This file contains:

Supplementary Table S1, S2, S3, S4, S8, S9, S10, S11, S12, S15

Supplementary Figure S1, S2. Original Plots of Western blotting

.

Table S1 Comparison of similarities and differences between different cell death types

	Cell death types	Apoptosis	Autophagy	Necrosis	Pyroptosis
**Similarities**	All are types of programmed cell death				
**Differences**	Incentives	Physiological	Pathological stimulus	Pathological stimulus	Pathological stimulus
	Cellular morphology	Shrink, apoptotic vesicles	Cavitation	Expansion, deformation	Expansion, deformation
	Cell membrane	Incomplete	Incomplete	Fracture	Fracture
	Organelle	Incomplete	Consumed by autophagus and lysosomes	Enlargement, deformation	Deformation
	DNA degradation	180-200 bp and integer multiples thereof	Stochastic degradation	Stochastic degradation	Stochastic degradation

Table S2　Characterization of different cell death types in UC epithelial cells

Cell death types	Changes in UC	Role in UC	Key mechanisms
**Apoptosis**	Increase	Disrupting the intestinal barrier and promotes intestinal inflammation.	Heat stress proteins such as HSP60 lead to mitochondrial dysfunction [1-2]; ROS-mediated injury and other stressors [1]; Induction of inflammatory factors such as TNF [3].
**Autophagy**	Decrease	Maintenance of intestinal barrier function is inhibited.	Defective autophagy leading to sustained endoplasmic reticulum stress and ROS-induced oxidative stress [4].
**Necrosis**	Increase	Disrupting the intestinal barrier and promotes intestinal inflammation.	Activating the mTOR-RIPK3 pathway [5]. Overactivating the RIPK1/ RIPK3/MLKL signaling axis [6]. Oxidative stress-induced mislocalization of nuclear RNA/DNA-binding proteins causing R-loop accumulation and activation of cell necrosis due to NAD+ depletion [7].
**Pyroptosis**	Increase	Promoting intestinal inflammation.	The NLRP3/casepse-1/GSDMD pathway is activated to release IL-1β and IL-18 pro-inflammatory factors [8].

References

1. Wang, M. et al. Precisely Inhibiting Excessive Intestinal Epithelial Cell Apoptosis to Efficiently Treat Inflammatory Bowel Disease with Oral Pifithrin-α Embedded Nanomedicine (OPEN). Advanced Materials 35, 2309370 (2023).
2. Berger, E. et al. Mitochondrial function controls intestinal epithelial stemness and proliferation. Nat. Commun. 7, 13171 (2016).
3. Wu, J. et al. EFHD2 suppresses intestinal inflammation by blocking intestinal epithelial cell TNFR1 internalization and cell death. Nat. Commun. 15, 1282 (2024).
4. Liu, M. et al. BRG1 attenuates colonic inflammation and tumorigenesis through autophagy-dependent oxidative stress sequestration. Nan. Commun. 10, 4614 (2019).
5. Xie, Y. et al. Gut epithelial TSC1/mTOR controls RIPK3-dependent necroptosis in intestinal inflammation and cancer. Journal of Clinical Investigation 130, 2111–2128 (2020).
6. Xu, J. et al. Epithelial Gab1 calibrates RIPK3-dependent necroptosis to prevent intestinal inflammation. JCI. Insight 8, e162701 (2023).
7. Yang, X. et al. Excessive nucleic acid R-loops induce mitochondria-dependent epithelial cell necroptosis and drive spontaneous intestinal inflammation. Proc. Natl. Acad. Sci. U.S.A. 121, e2307395120 (2024).
8. Liu, W. et al. Ablation of caspase-1 protects against TBI-induced pyroptosis in vitro and in vivo. J . Neuroinflammation 15, 48 (2018).

Table S3 Characterization of different cell death types in UC immune cells

Cell death types	Changes in UC	Role in UC	Key mechanisms
**Apoptosis**	Downregulated in neutrophils	Inhibiting inflammation	CARD9 inhibits mitochondrial hyperactivation, protects its function, and inhibits the production of mtROS [1].
**Autophagy**	Upregulated in neutrophils	Promoting inflammation	Upregulation of Sky expression activates the mTOR/RUBCNL pathway-mediated inflammatory response [2].
**Necrosis**	Downregulated in neutrophils and macrophages	Inhibiting inflammation	Tim-3 downregulates TLR4/NF-κB and RIPI/RIP3 pathways and inhibits ROS generation [3].
**Pyroptosis**	Upregulated in macrophages	Inhibiting inflammation	GSDMD in macrophages negatively regulates the cGAS-STING pathway-mediated inflammatory response[4].
		Promoting inflammation (major)	NLRP3/caspase-1 pathway is activated and release IL-1β and IL-18 pro-inflammatory factors[5].

References

1. Danne, C. et al. CARD9 in neutrophils protects from colitis and controls mitochondrial metabolism and cell survival. Gut 72, 1081–1092 (2023).
2. Zhu, F. et al. Blockade of Syk modulates neutrophil immune-responses via the mTOR/RUBCNL-dependent autophagy pathway to alleviate intestinal inflammation in ulcerative colitis. Precision Clinical Medicine 6, pbad.025 (2023).
3. Wang, F. et al. Macrophage Tim-3 maintains intestinal homeostasis in DSS-induced colitis by suppressing neutrophil necroptosis. Redox Biology 70, 103072 (2024).
4. Ma, C. et al. Gasdermin D in macrophages restrains colitis by controlling cGAS-mediated inflammation. Sci. Adv. 6, eaaz6717 (2020).
5. Gong, W. et al. Mincle/Syk Signalling Promotes Intestinal Mucosal Inflammation Through Induction of Macrophage Pyroptosisin Crohn's Disease. Journal of Crohn's Disease and Colitis 14, 1734 – 1747 (2020).

Table S4 Functions of Disulfidptosis-related genes in the disulfidptosis

Disulfidptosis-related genes	Function
SCL7A11	The overexpression of the SLC7A11 leads to accumulation of disulfides such as cystine and γ-glutamyl-cystine, causing disulfide stress and promoting disulfidptosis [1-3].
SLC3A2	SLC3A2 constitutes a glutamate-cystine antitransporter system with SLC7A11 and acts synergistically in promoting disulfidptosis [1-3].
NCKAP1、WASF2、CYFIP1 ABI2、BRK1	They co-encode five subunits that constitute the WAVE regulatory complex WAC, which promotes actin polymerization and lamellar pseudopod formation, and deletion of any of these subunits attenuates disulfidptosis. Activation of its upstream activator molecule Rac1 also promotes disulfidptosis [3-5].
NUBPL、NDUFA11、NDUFS1 LRPPRC、OXSM、GYS1	They are involved in mitochondrial oxidative phosphorylation and glycogen synthesis, up-regulated expression inhibits disulfidptosis [3].
FLNA、FLNB、MYL6、MYH9 CAPZB、CD2AP 、DSTN、TLN1、MYH10、ACTB、ACTN、INF2、PDLIM1 、IQGAP1	They encodes cytoskeletal actin, which increases disulfide bond formation under glucose starvation conditions. F-actin regulates cytoskeletal contract, causing disulfide bonds rupture, which results in separation of the cytoskeleton from the cell membrane, leading to cell death [3].
RPN1	The upregulating expression of RPN1 promotes disulfidptosis [3].

References

1. Lin, W. *et al.* SLC7A11/xCT in cancer: biological functions and therapeutic implications. *Am . J . Cance. Re.* **10**, 3106–3126 (2020).
2. Koppula, P., Zhang, Y., Zhuang, L. & Gan, B. Amino acid transporter SLC7A11/xCT at the crossroads of regulating redox homeostasis and nutrient dependency of cancer. *Cancer Communications* **38**, 1–13 (2018).
3. Liu, X. *et al.* Actin cytoskeleton vulnerability to disulfide stress mediates disulfidptosis. *Nat. Cell Bio.* **25**, 404–414 (2023).
4. Miki, H., Suetsugu, S. & Takenawa, T. WAVE, a novel WASP-family protein involved in actin reorganization induced by Rac. *EMBO J .* **17**, 6932–6941 (1998).
5. Steffen, A. *et al.* Sra-1 and Nap1 link Rac to actin assembly driving lamellipodia formation. *EMBO J .***23**, 749–759 (2004).

Table S8: The correlation analysis of candidate genes

Gene_1	Gene_2	cor	p.value
SLC7A11	FLNA	0.284443001	0.010064829
SLC7A11	MYH10	0.205218181	0.066081509
SLC7A11	NUBPL	-0.147130575	0.189945669
SLC7A11	LRPPRC	-0.061967331	0.582616913
SLC7A11	NDUFS1	-0.208878718	0.061291159
SLC7A11	CD2AP	-0.002843412	0.97990102
FLNA	MYH10	0.810473175	4.91E-20
FLNA	NUBPL	-0.20169994	0.070966492
FLNA	LRPPRC	-0.220199229	0.048233217
FLNA	NDUFS1	-0.59797247	3.76E-09
FLNA	CD2AP	-0.77607246	1.74E-17
MYH10	NUBPL	-0.023953818	0.831901074
MYH10	LRPPRC	-0.108339263	0.335680132
MYH10	NDUFS1	-0.406240081	0.000167906
MYH10	CD2AP	-0.684361232	1.87E-12
NUBPL	LRPPRC	0.838151014	1.69E-22
NUBPL	NDUFS1	0.77661381	1.60E-17
NUBPL	CD2AP	0.251105156	0.023748052
LRPPRC	NDUFS1	0.776557554	1.62E-17
LRPPRC	CD2AP	0.297982864	0.006895795
NDUFS1	CD2AP	0.629583483	3.06E-10

Table S9 The list of characterized genes screened by least absolute shrinkage and selection operator

lasso_fea	myCoefs.x
SLC7A11	1.45404806
LRPPRC	-2.278102963
NDUFS1	-0.737542333
CD2AP	-3.828453783

Table S10 The list of characterized genes screened by support vector machines

Feature Name	Feature ID	AvgRank
CD2AP	7	1
SLC7A11	1	2.8
FLNA	2	4
NUBPL	4	4.2
LRPPRC	5	4.8
MYH10	3	5.4
NDUFS1	6	5.8

Table S11 ROC curve analysis of characterized genes in the training set

	group	CD2AP	NDUFS1	LRPPRC	SLC7A11
GSM1187610	UC	10.4248536	8.914733187	9.259106123	9.807153942
GSM1187611	UC	10.2798217	8.627491107	8.77866927	9.889955699
GSM1187612	UC	9.92924285	8.86077639	9.059260378	9.380214052
GSM1187613	UC	9.62617968	8.751541767	9.041556119	10.11477436
GSM1187614	UC	10.1330835	8.952773834	9.264434424	9.200630015
GSM1187615	UC	10.4071411	8.792515031	8.866930005	10.55165869
GSM1187616	UC	10.0432367	8.67154933	8.423382344	8.838271296
GSM1426090	UC	10.6720387	8.942792346	9.105861658	8.248784662
GSM1426091	UC	10.673871	9.113219454	8.974182307	7.871120805
GSM1426092	UC	10.8149814	9.319299587	9.050472408	7.221653988
GSM1426093	UC	10.7453258	8.924374186	8.917855594	8.582632301
GSM1426094	UC	8.83564503	8.362628524	8.354857292	6.912331282
GSM1426095	UC	9.74286281	8.732406281	8.809777926	8.611750325
GSM1426096	UC	10.530126	8.766362171	8.960065111	9.264210691
GSM1426097	UC	10.5672712	8.857251471	8.884060408	8.756455507
GSM1426098	UC	10.8890788	8.373158117	8.151107412	9.73147823
GSM1426099	UC	10.6535475	9.578730715	9.847772234	9.634708423
GSM1426100	UC	10.005023	8.797173379	9.015546154	8.868544772
GSM1426101	UC	10.1995957	8.537039566	9.415671397	9.54601256
GSM1426102	UC	10.7482771	9.200240868	9.48315224	8.382818993
GSM1426103	UC	10.7732025	9.138245857	9.141283855	9.562680756
GSM1426104	UC	10.4909571	9.199846872	9.425054611	9.855323251
GSM1426105	UC	10.273647	8.92278411	9.155332964	8.900604139
GSM1426106	UC	10.5830874	9.458570197	9.644541173	8.913414255
GSM1426107	UC	10.496226	9.011619446	9.095991328	9.412686376
GSM1426108	UC	11.0045855	9.411634796	9.464849183	9.560306601
GSM1426109	UC	10.4320647	9.125622419	9.178612049	9.20764855
GSM1426110	UC	10.7565042	9.83137641	9.837171447	7.82544943
GSM1426111	UC	10.66136	9.152246583	9.203674966	8.81319887
GSM1426112	UC	10.3417978	9.350824691	9.458936406	8.844799548

GSM1426113	UC	10.2326563	9.149649452	9.519052282	9.015879295
GSM1426114	UC	10.0149112	8.749435587	8.909040699	9.620544486
GSM1426115	UC	10.4138907	8.811838043	9.181432265	8.419291054
GSM1426116	UC	9.98320881	9.097150173	9.322126182	9.1133948
GSM1426117	UC	10.6673568	9.24730954	9.493789927	8.76233318
GSM1426118	UC	10.2508199	9.159972203	9.335526923	8.324537378
GSM1426119	UC	10.3226279	8.988410817	8.837636739	7.773862243
GSM1426120	UC	10.4126022	9.286219336	9.473886121	8.167792091
GSM1426121	UC	10.6526917	9.137198252	9.185044666	9.563558708
GSM1426122	UC	10.5327931	9.048976027	9.071485768	8.890795473
GSM1426123	UC	10.8463074	8.751890901	8.619742529	10.35064588
GSM1426124	UC	10.4487309	9.128905774	9.62491803	8.4328832
GSM1426125	UC	10.5691046	8.885363385	8.963235972	8.557679712
GSM1426126	UC	9.50672438	8.486754477	8.726066902	8.976375755
GSM1426127	UC	10.5120372	8.936524765	9.000223092	8.79446808
GSM1426128	UC	10.3083991	8.873237853	9.061033229	8.771501771
GSM1426129	UC	10.6106615	8.948126716	9.322218677	9.443484117
GSM1426130	UC	10.557589	8.834009531	8.771677512	7.280479574
GSM1426131	UC	10.0000036	8.664939325	9.209627842	7.891013041
GSM1426132	UC	9.25264679	8.628887736	8.905886364	8.522264529
GSM1426133	UC	10.3907659	9.238477628	9.37561904	8.594843949
GSM1426134	UC	10.4157359	9.001268302	9.112123415	9.390856533
GSM1426135	UC	10.6075949	9.260935463	9.696196913	8.429811772
GSM1426136	UC	10.6269891	8.867324275	8.351366536	8.995086161
GSM1426137	UC	11.1553573	8.934472335	8.388240407	9.389144209
GSM1426138	UC	10.4434	9.122959633	9.225961442	9.029131981
GSM1426139	UC	9.82155733	8.882586646	9.204403628	8.524726854
GSM1426140	UC	10.2572491	8.945146702	9.048071638	9.04964657
GSM1426141	UC	10.7120082	9.07778184	9.106331295	7.563724689
GSM1426142	UC	9.94012312	9.037154408	9.119186191	8.568105881
GSM1426143	UC	10.4730584	9.201209551	9.271026152	6.418996845
GSM1426144	UC	10.8385081	8.950372419	8.707456134	9.054867634

GSM1426145	UC	9.9680024	9.028636626	9.523810032	9.445418096
GSM1426146	UC	10.3289409	8.844126898	9.09673449	9.014986394
GSM1426147	UC	10.4121295	9.074703804	9.222273372	8.118592084
GSM1426148	UC	10.7000162	9.207308735	9.185397876	7.070732359
GSM1426149	UC	10.3068252	9.116428704	9.170359559	8.230650254
GSM1426150	UC	10.3183316	8.844764579	9.095883103	8.640165606
GSM1426151	UC	10.6162071	9.172104096	9.093569277	7.341602796
GSM1426152	UC	10.2531645	8.819517347	9.192937412	7.904989018
GSM1426153	UC	10.2914379	8.994155122	9.012356386	8.073948388
GSM1426154	UC	10.6189149	9.141217841	9.215209723	7.955569233
GSM1426155	UC	10.7086245	9.079811141	8.90015961	7.898937312
GSM1426156	UC	10.6601085	9.357880202	9.526504353	8.700268154
GSM1426157	UC	10.737271	9.320282947	9.408761964	7.297740842
GSM1426158	UC	8.60926519	7.88984394	7.977313129	8.802190821
GSM1426159	UC	9.75484488	8.651755796	8.786931537	8.860133185
GSM1426160	UC	10.1281859	8.830895357	9.155028006	9.633556148
GSM1426161	UC	10.5754425	8.847231318	8.522534144	8.553129283
GSM1426162	UC	10.1497127	8.80798798	9.166247482	8.814720188
GSM1426163	UC	10.7318515	9.204393115	9.37648912	8.184325025
GSM1187602	Control	11.111938	9.382189032	9.329703577	6.946805
GSM1187603	Control	10.9540154	9.538188821	9.633929264	6.863866422
GSM1187604	Control	11.0024279	9.769152428	9.610321634	7.40008125
GSM1187605	Control	10.7210626	9.229932116	9.406576911	7.131101399
GSM1187606	Control	10.8391921	9.563096855	9.500215083	7.867772531
GSM1187607	Control	10.9674985	9.56490341	9.703588221	7.329348294
GSM1187608	Control	11.0210266	9.602938602	9.818382024	7.093200246
GSM1187609	Control	11.0754027	9.310348403	9.482562739	7.626240222
GSM1426079	Control	10.7347399	9.5310346	9.636154969	6.256476226
GSM1426080	Control	10.8478545	9.523171323	10.02407601	6.801543449
GSM1426081	Control	11.1575649	9.693643403	10.07590352	6.534784552
GSM1426082	Control	11.2511368	9.821370787	9.856839499	6.627322163
GSM1426083	Control	10.6729485	9.882245466	9.842930948	6.300203651

GSM1426084	Control	10.8776139	9.800457508	9.959945341	6.446388782
GSM1426085	Control	10.9014492	9.501109941	9.604809157	5.990657336
GSM1426086	Control	11.2159215	9.593018609	9.668146635	6.529605057
GSM1426087	Control	11.1723911	9.827433198	9.904490507	5.957905754
GSM1426088	Control	11.1201305	9.780351838	9.780316309	5.752440826
GSM1426089	Control	11.2407597	9.651690505	9.552776123	5.830074554

Table S12 ROC curve analysis of characterized genes in the validation set

	group	CD2AP	LRPPRC	NDUFS1	SLC7A11
GSM2332079	UC	7.3015	6.234525	7.49116	7.994133333
GSM2332080	UC	7.15585	6.496575	7.0841	6.904033333
GSM2332081	UC	7.85655	6.114275	7.68482	7.424233333
GSM2332082	UC	7.30845	5.97375	6.6508	6.494633333
GSM2332083	UC	6.9696	6.3875	6.78266	6.6811
GSM2332084	UC	7.93215	5.333525	6.87494	8.494433333
GSM2332085	UC	7.035	6.680025	7.58762	5.791566667
GSM2332086	UC	7.0658	6.673575	7.206	6.1672
GSM2332087	UC	6.84475	6.314675	6.95762	6.848833333
GSM2332088	UC	6.8132	6.5344	7.2538	6.459233333
GSM2332089	UC	7.141	7.361625	8.05064	4.979066667
GSM2332090	UC	7.1084	6.722175	7.53844	5.274633333
GSM2332091	UC	7.12275	6.413675	7.39812	6.620766667
GSM2332092	UC	7.5286	6.409175	7.46518	7.875433333
GSM2332093	UC	7.1663	7.05415	7.98572	5.127966667
GSM2332094	UC	7.6558	6.370975	7.00852	7.104
GSM2332095	UC	7.48245	6.854875	8.16192	5.1763
GSM2332096	UC	6.8609	6.830475	7.14862	6.367266667
GSM2332097	UC	7.16945	6.309825	7.09382	5.683366667
GSM2332098	Control	8.1205	9.3158	6.3832	5.5254
GSM2332099	Control	8.04	8.504575	6.30652	4.7763
GSM2332100	Control	8.2517	9.31855	6.37288	4.966633333
GSM2332101	Control	8.27875	9.4496	6.4307	5.368666667
GSM2332102	Control	8.29485	9.712	6.18386	5.426766667
GSM2332103	Control	8.53915	9.3645	6.08406	4.717766667
GSM2332104	Control	8.47555	8.923225	6.07016	4.733733333
GSM2332105	Control	8.23605	9.0683	5.9897	5.6044
GSM2332106	Control	8.01105	8.6583	5.82662	4.743
GSM2332107	Control	8.4587	9.066975	6.06726	5.334933333
GSM2332108	Control	8.26955	8.9613	6.2165	6.0434

GSM2332109	Control	8.19995	8.88095	6.27166	5.2275
GSM2332110	Control	8.03635	8.371275	6.1847	4.827666667
GSM2332111	Control	8.0969	9.316525	6.1148	5.390233333
GSM2332112	Control	8.1068	9.4378	6.39046	6.285533333
GSM2332113	Control	8.14545	9.057725	6.35658	4.9956
GSM2332114	Control	8.36815	9.28115	6.42226	5.3163
GSM2332115	Control	8.4629	8.796225	5.99904	4.8221
GSM2332116	Control	8.3585	9.270025	6.23316	5.597966667
GSM2332117	Control	8.4469	9.32995	6.50006	5.759833333
GSM2332118	Control	8.09405	9.188975	6.28814	5.220166667
GSM2332119	UC	7.99595	8.931925	5.8954	6.114666667
GSM2332120	UC	7.9312	8.59335	5.83874	6.835266667
GSM2332121	UC	8.13185	8.62205	5.9673	6.489133333
GSM2332122	UC	7.9713	8.433775	5.63552	5.461466667
GSM2332123	UC	7.8842	8.333025	5.70164	6.4573
GSM2332124	UC	7.8426	8.834675	5.94036	6.933
GSM2332125	UC	8.0333	8.860375	6.40008	5.188466667
GSM2332126	UC	7.9039	8.90325	6.10894	6.649466667
GSM2332127	UC	7.9689	9.0104	6.49674	6.311066667
GSM2332128	UC	7.8165	8.5517	5.9041	7.010533333
GSM2332129	UC	8.1395	8.6845	6.1013	5.791166667
GSM2332130	UC	7.7788	8.873775	5.75408	6.975733333
GSM2332131	UC	8.0202	8.877725	6.12436	6.413433333
GSM2332132	UC	8.23825	9.144375	6.1725	4.844733333
GSM2332133	UC	7.8459	8.68355	5.8443	7.4555
GSM2332134	UC	8.26755	8.5812	5.92274	7.404966667
GSM2332135	UC	7.80045	8.5485	5.64984	7.4165
GSM2332136	UC	8.0449	9.1402	6.27488	6.369766667
GSM2332137	UC	7.95765	8.614275	6.06478	6.2674
GSM2332138	UC	7.97865	8.639225	5.91412	5.9727
GSM2332139	UC	7.9735	8.621775	6.11074	6.2478
GSM2332140	UC	7.89275	9.099425	6.0446	6.019833333

GSM2332141	UC	7.87505	8.6601	5.82464	5.802966667
GSM2332142	UC	7.7633	8.465375	5.62436	6.716933333
GSM2332143	UC	7.84455	7.92695	5.6051	5.4919
GSM2332144	UC	7.3957	8.600225	5.55234	8.228033333
GSM2332145	UC	8.121	8.5422	5.72864	6.640066667
GSM2332146	UC	8.05795	9.1632	6.11286	4.7334
GSM2332147	UC	8.2845	8.13505	6.05298	7.277433333
GSM2332148	UC	8.1051	8.61465	5.85794	7.4775
GSM2332149	UC	8.16705	9.1617	6.39966	5.272633333
GSM2332150	UC	8.12375	8.951	5.96882	6.218833333
GSM2332151	UC	8.1058	8.846475	5.88594	6.009833333
GSM2332152	UC	7.65725	8.29065	5.7846	7.174533333
GSM2332153	UC	8.09235	8.57785	5.93694	6.369366667
GSM2332154	UC	8.0069	8.483725	5.81694	6.779833333
GSM2332155	UC	8.2135	8.17895	5.63746	6.798066667
GSM2332156	UC	7.9512	8.604925	5.73952	5.767
GSM2332157	UC	7.9652	8.469	5.93256	7.0814
GSM2332158	UC	7.62695	8.29815	5.6776	7.011
GSM2332159	UC	7.75785	8.58005	5.71846	6.764266667
GSM2332160	UC	8.1751	8.190375	5.718	6.1017
GSM2332161	UC	7.8783	8.617475	5.67692	6.384766667
GSM2332162	UC	7.81425	8.4589	5.7304	7.158533333
GSM2332163	UC	7.76345	8.458	5.56806	6.198366667
GSM2332164	UC	7.8066	8.5236	5.6497	6.869733333
GSM2332165	UC	7.82475	8.462725	5.56668	6.435166667
GSM2332166	UC	8.01925	8.665625	6.06652	6.336433333
GSM2332167	UC	7.83655	8.589325	5.67506	5.842633333
GSM2332168	UC	7.93715	8.554775	5.72988	7.9638
GSM2332169	UC	8.08795	8.9148	6.0227	6.836833333
GSM2332170	UC	7.94475	8.5377	5.59456	6.071666667
GSM2332171	UC	7.59555	8.3629	5.52424	6.853133333
GSM2332172	UC	8.15195	8.94705	6.06184	6.659566667

GSM2332173	UC	8.11425	9.0595	6.02812	5.022966667
GSM2332174	UC	7.59355	8.41545	5.7298	6.507666667
GSM2332175	UC	7.8966	9.219875	6.0064	7.081
GSM2332176	UC	8.1119	8.9613	6.12314	6.250733333
GSM2332177	UC	8.1381	8.85655	6.00036	5.782566667
GSM2332178	UC	7.755	8.68125	5.66144	6.6002
GSM2332179	UC	7.73975	8.486375	5.58134	6.2203
GSM2332180	UC	8.2562	8.485825	6.14196	6.3401
GSM2332181	UC	8.09075	8.323075	5.91616	5.9561
GSM2332182	UC	7.7594	8.76755	5.88294	7.756766667
GSM2332183	UC	7.74345	8.83625	5.91176	5.597166667
GSM2332184	UC	8.0669	8.802775	6.18622	5.808466667
GSM2332185	UC	8.09835	8.7764	5.99768	7.814733333
GSM2332186	UC	8.1727	8.413325	5.83162	6.527066667
GSM2332187	UC	8.0861	8.363575	6.00574	5.972533333
GSM2332188	UC	8.25385	8.338175	5.96732	6.306333333
GSM2332189	UC	7.98055	8.420225	5.94058	6.9422
GSM2332190	UC	8.09505	8.74335	6.13244	6.147466667
GSM2332191	UC	7.9596	8.631975	5.84556	5.7573
GSM2332192	UC	8.0751	8.714175	6.085	6.241066667
GSM2332193	UC	7.99755	8.687825	5.90462	7.043133333
GSM2332194	UC	7.91135	8.4662	5.73374	7.2986
GSM2332195	UC	7.97995	8.607675	5.61224	6.8603
GSM2332196	UC	8.19115	7.26525	5.48936	6.788
GSM2332197	UC	8.2041	8.716525	6.15558	6.4159
GSM2332198	UC	7.80365	8.74135	5.69612	7.0594
GSM2332199	UC	8.0469	8.0952	5.9256	7.043266667
GSM2332200	UC	8.01175	8.923425	5.89924	6.937633333
GSM2332201	UC	7.70225	8.328575	5.64476	7.867333333
GSM2332202	UC	7.72485	8.5967	5.70064	6.724766667
GSM2332203	UC	8.20615	8.502425	5.88024	6.960366667
GSM2332204	UC	7.83255	8.291275	5.62558	5.7812
GSM2332205	UC	8.1158	8.670175	5.9075	7.341133333

Table S15 Correlation analysis between characterized genes and immune cells

gene	Cell.type	Correlation coefficient	*p*.value
SLC7A11	B cells memory	-0.221870605	0.0481201
SLC7A11	T cells CD8	-0.322409752	0.0036841
SLC7A11	T cells CD4 naive	-0.172961473	0.1249669
SLC7A11	T cells CD4 memory resting	0.242856138	0.029962
SLC7A11	T cells CD4 memory activated	0.112725345	0.3194662
SLC7A11	T cells follicular helper	0.213087224	0.0577261
SLC7A11	T cells regulatory (Tregs)	-0.33216962	0.0026105
SLC7A11	NK cells resting	0.31612752	0.0044405
SLC7A11	NK cells activated	-0.328652133	0.0029162
SLC7A11	Macrophages M0	0.299554618	0.0071411
SLC7A11	Macrophages M1	0.29765834	0.0073291
SLC7A11	Macrophages M2	-0.448828862	2.97E-05
SLC7A11	Dendritic cells activated	0.297798276	0.0073002
SLC7A11	Mast cells resting	-0.33430216	0.0024396
SLC7A11	Mast cells activated	0.473059917	9.37E-06
SLC7A11	Eosinophils	-0.214378692	0.0561914
SLC7A11	Neutrophils	0.497872581	2.62E-06
LRPPRC	B cells memory	0.251922175	0.0244258
LRPPRC	T cells CD8	0.276793249	0.0131711
LRPPRC	T cells CD4 naive	0.182705781	0.1047741
LRPPRC	T cells CD4 memory resting	-0.069598443	0.5395758
LRPPRC	T cells CD4 memory activated	-0.16666116	0.1395264
LRPPRC	T cells follicular helper	0.006146865	0.956844
LRPPRC	T cells regulatory (Tregs)	0.075540778	0.5054265
LRPPRC	NK cells resting	-0.225082044	0.0449115
LRPPRC	NK cells activated	0.106736523	0.3460131
LRPPRC	Macrophages M0	-0.305555556	0.00603
LRPPRC	Macrophages M1	-0.078028195	0.4914708
LRPPRC	Macrophages M2	0.275667396	0.0133237
LRPPRC	Dendritic cells activated	-0.05518082	0.6268572
LRPPRC	Mast cells resting	0.121690454	0.2822384
LRPPRC	Mast cells activated	-0.120384846	0.2874726
LRPPRC	Eosinophils	0.289262274	0.0092571
LRPPRC	Neutrophils	-0.220204615	0.0496768
NDUFS1	B cells memory	0.171308017	0.1285145
NDUFS1	T cells CD8	0.406516643	0.0002067
NDUFS1	T cells CD4 naive	0.177833627	0.1145274
NDUFS1	T cells CD4 memory resting	-0.274830634	0.0136182
NDUFS1	T cells CD4 memory activated	-0.308673551	0.0053399
NDUFS1	T cells follicular helper	0.064382265	0.570457
NDUFS1	T cells regulatory (Tregs)	0.331498742	0.0026665
NDUFS1	NK cells resting	-0.334130333	0.0025743
NDUFS1	NK cells activated	0.210208588	0.0612701
NDUFS1	Macrophages M0	-0.299484294	0.0071551
NDUFS1	Macrophages M1	-0.29754111	0.0073534
NDUFS1	Macrophages M2	0.329596171	0.0028311
NDUFS1	Dendritic cells activated	-0.119667837	0.2903742
NDUFS1	Mast cells resting	0.22467806	0.0451076
NDUFS1	Mast cells activated	-0.202941857	0.0710064

NDUFS1	Eosinophils	0.30924778	0.0052508
NDUFS1	Neutrophils	-0.375646675	0.0005955
CD2AP	B cells memory	0.030332865	0.7890078
CD2AP	T cells CD8	0.411157993	0.0001725
CD2AP	T cells CD4 naive	0.177833627	0.1145274
CD2AP	T cells CD4 memory resting	-0.244965892	0.0285211
CD2AP	T cells CD4 memory activated	-0.381555335	0.0004792
CD2AP	T cells follicular helper	-0.12454778	0.2710058
CD2AP	T cells regulatory (Tregs)	0.361310087	0.000992
CD2AP	NK cells resting	-0.316549461	0.0043857
CD2AP	NK cells activated	0.240331657	0.0317662
CD2AP	Macrophages M0	-0.273722457	0.0142554
CD2AP	Macrophages M1	-0.342487039	0.0018727
CD2AP	Macrophages M2	0.269054491	0.0158086
CD2AP	Dendritic cells activated	-0.115811743	0.3063097
CD2AP	Mastcells resting	0.230390323	0.0397836
CD2AP	Mastcells activated	-0.293201048	0.0083034
CD2AP	Eosinophils	0.216116562	0.054179
CD2AP	Neutrophils	-0.32174944	0.0036104

Original Plots of Western blotting

In our Western blotting procedures, blots were cut according to the molecular weights of the target proteins prior to antibody hybridization. Consequently, we are unable to provide images of full-length membranes at this time. As requested, we uploaded blots images of all target proteins for both human and animal samples.

The Marker used in this study was PageRuler Prestained Protein Ladder, 10 to 180 kDa (Thermo Fisher)

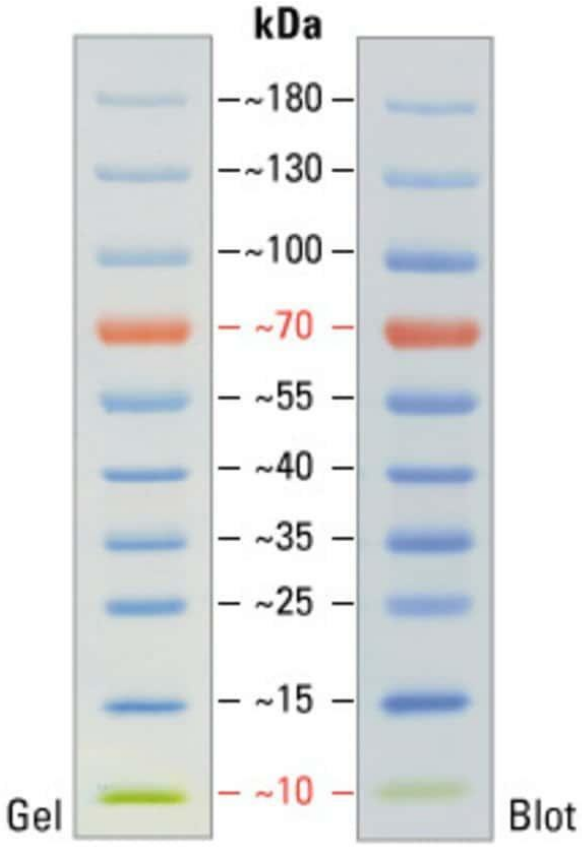

Supplementary Figure S1

(1) The protein expression levels of the characterized genes in the intestinal mucosal tissues of normal control mice and DSS-induced colitis mice were examined by western blotting, and the original images are shown in the following figures. These figures correspond to Figure 8e in the manuscript. (Definition of abbreviations: normal control mice(N=6), DSS induced colitis mice (DSS), (N=6))

Related to Fig.8e

SLC7A11 (55kDa)	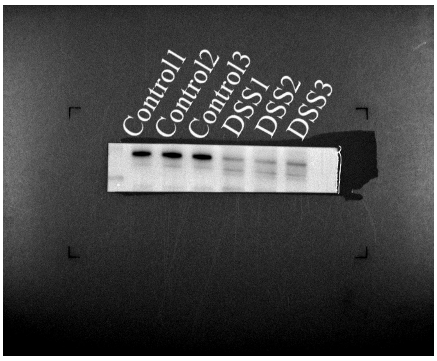	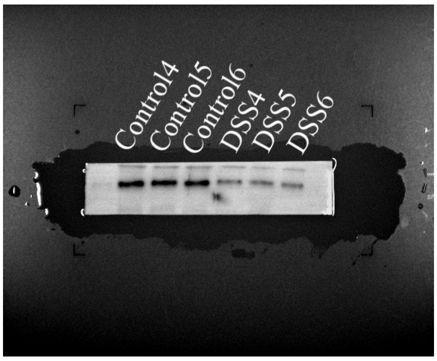
LRPPRC	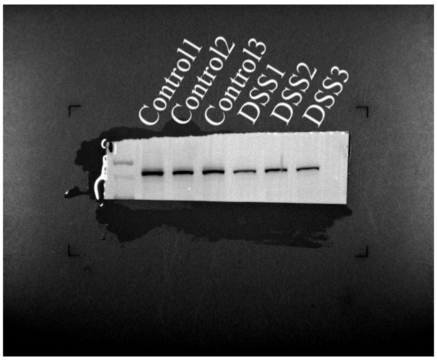	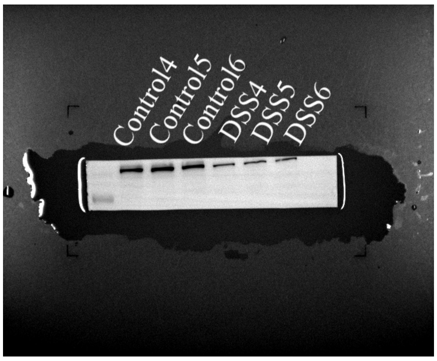
NDUFS1	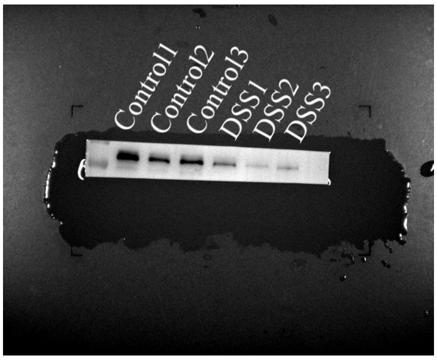	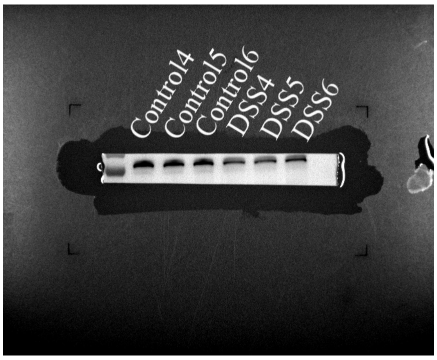

<table>
<tr>
<td>CD2AP
(56kDa)</td>
<td>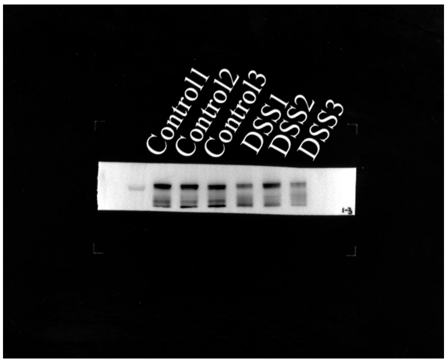
</td>
<td>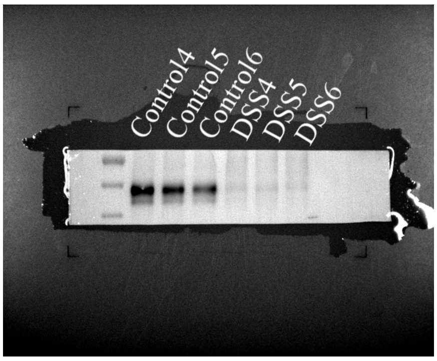
</td>
</tr>
<tr>
<td>Vinculin
(130kDa)</td>
<td>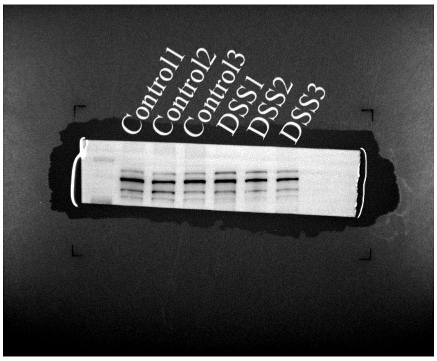
</td>
<td>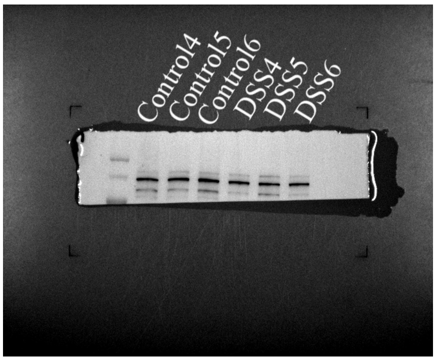
</td>
</tr>
</table>

Supplementary Figure S2

(2) The protein expression levels of the characterized genes in the intestinal mucosal tissues of healthy individuals and ulcerative colitis patients were examined by western blotting, and the original images are shown in the following figures. These figures correspond to Figure 8g in the manuscript. (Definition of abbreviations: healthy control (HC, N=5), ulcerative colitis (UC, N=5))

(Related to Fig.8g)

<table>
<tr><td>NDUFS1
(79 kDa)</td><td>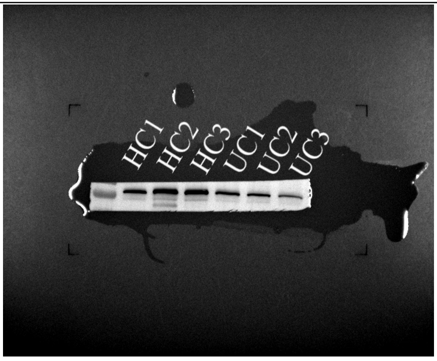
</td><td>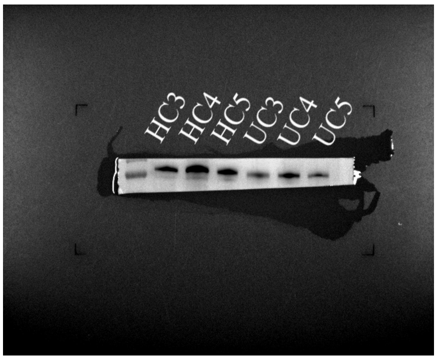
</td></tr>
<tr><td>LRPPRC
(130kDa)</td><td>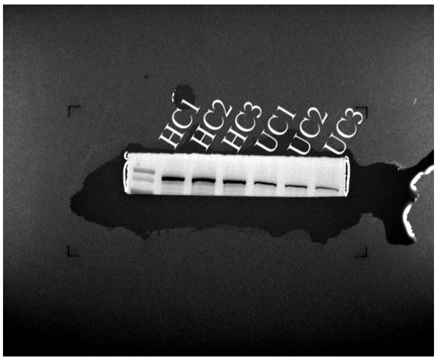
</td><td>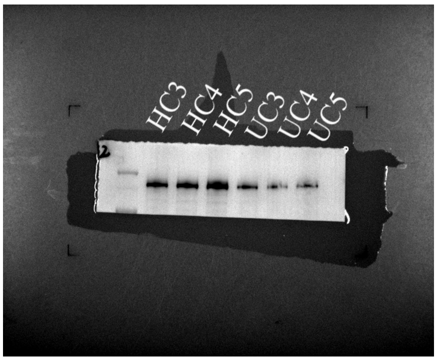
</td></tr>
<tr><td>SLC7A11
(55 kDa)</td><td>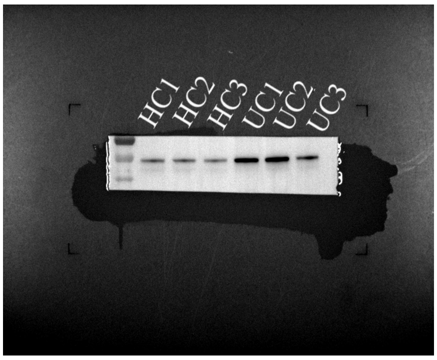
</td><td>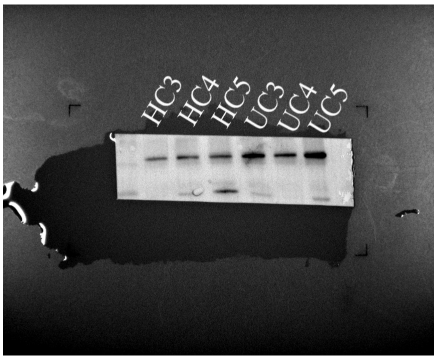
</td></tr>
</table>

<table>
<tr><td>CD2AP
(56kDa)</td><td>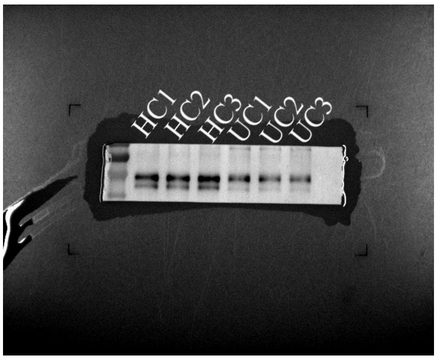
</td><td>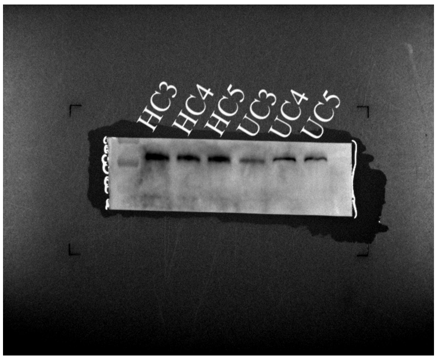
</td></tr>
<tr><td></td><td></td><td></td></tr>
<tr><td>Vinculin
(56 kDa)</td><td>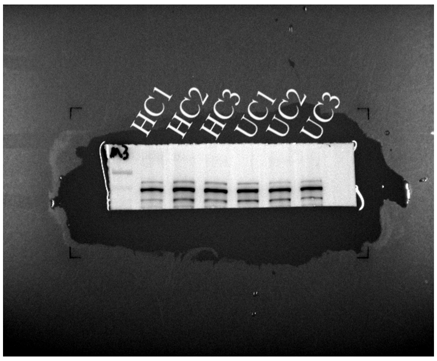
</td><td>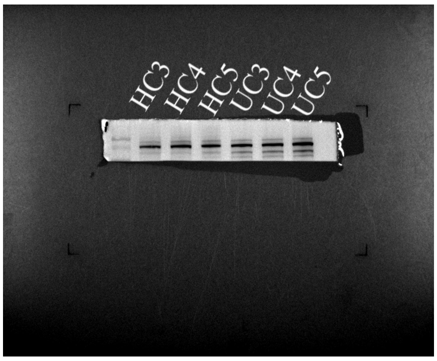
</td></tr>
</table>
